# Non-contrast and contrast enhanced computed tomography radiomics in preoperative discrimination of lung invasive and non-invasive adenocarcinoma

**DOI:** 10.3389/fmed.2022.939434

**Published:** 2022-11-04

**Authors:** Yingli Sun, Wei Zhao, Kaiming Kuang, Liang Jin, Pan Gao, Shaofeng Duan, Yi Xiao, Jun Liu, Ming Li

**Affiliations:** ^1^Department of Radiology, Huadong Hospital Affiliated to Fudan University, Shanghai, China; ^2^Department of Radiology, Second Xiangya Hospital, Central South University, Changsha, China; ^3^Dianei Technology, Shanghai, China; ^4^GE Healthcare, Shanghai, China; ^5^Department of Radiology, Changzheng Hospital, Second Military Medical University, Shanghai, China

**Keywords:** adenocarcinoma, lung, radiomics, solitary pulmonary nodule, X-ray computed tomography

## Abstract

**Objective:**

This study aimed to assess the value of radiomics based on non-contrast computed tomography (NCCT) and contrast-enhanced computed tomography (CECT) images in the preoperative discrimination between lung invasive adenocarcinomas (IAC) and non-invasive adenocarcinomas (non-IAC).

**Methods:**

We enrolled 1,185 pulmonary nodules (478 non-IACs and 707 IACs) to build and validate radiomics models. An external testing set comprising 63 pulmonary nodules was collected to verify the generalization of the models. Radiomic features were extracted from both NCCT and CECT images. The predictive performance of radiomics models in the validation and external testing sets were evaluated and compared with radiologists’ evaluations. The predictive performances of the radiomics models were also compared between three subgroups in the validation set (Group 1: solid nodules, Group 2: part-solid nodules, and Group 3: pure ground-glass nodules).

**Results:**

The NCCT, CECT, and combined models showed good ability to discriminate between IAC and non-IAC [respective areas under the curve (AUCs): validation set = 0.91, 0.90, and 0.91; Group 1 = 0.82, 0.79, and 0.81; Group 2 = 0.93, 0.92, and 0.93; and Group 3 = 0.90, 0.90, and 0.89]. In the external testing set, the AUC of the three models were 0.89, 0.91, and 0.89, respectively. The accuracies of these three models were comparable to those of the senior radiologist and better those that of the junior radiologist.

**Conclusion:**

Radiomic models based on CT images showed good predictive performance in discriminating between lung IAC and non-IAC, especially in part solid nodule group. However, radiomics based on CECT images provided no additional value compared to NCCT images.

## Introduction

Lung cancer is the most commonly diagnosed cancer and the leading cause of cancer-related deaths worldwide (1). Despite the recent development of targeted therapies for selected sub-types of lung adenocarcinoma, the overall cure and survival rates for this cancer remain relatively low (2). Adenocarcinoma is the most common form of lung cancer and has recently been classified into pre-invasive adenocarcinoma [atypical adenocarcinoma hyperplasia (AAH), adenocarcinoma *in situ* (AIS)], minimally invasive adenocarcinoma (MIA), and invasive adenocarcinoma (IAC) (3). The 5-year disease-free survival rates in AIS and MIA are 100% or close to 100%, which are significantly higher than that in IAC (38–86%, depending on the predominant histological subtypes) (4, 5). Therefore, the accurate preoperative diagnosis of lung adenocarcinoma is critical for clinical decision-making processes and the assessment of prognoses.

Due to the diversity and overlap of radiographic features of these lesions, diagnosing and differentiating lung IAC is challenging for radiologists. Radiomics is an emerging method that can extract many features to facilitate the precision medicine (6). Many studies have explored the value of radiomics in the detection, characterization, and monitoring of lung nodules, resulting in promising performance (7–9). However, those studies focused on the radiomic features extracted from non-contrast CT (NCCT) images. The National Comprehensive Cancer Network (NCCN) recommends contrast-enhanced CT (CECT) examinations for some lung nodules: solid nodules > 15 mm on initial screening, part solid nodules with solid components > 8 mm in initial screening, new or increased solid nodules ≥ 8 mm during the follow-up, new or increased part-solid nodules with solid components > 1.5 mm during the follow-up) (10). The CECT images can yield better vascular information and improve the accuracy of the diagnoses. Several studies have assessed the value of radiomics based on CECT images in the diagnosis of pulmonary nodules (9, 11–14), but their conclusions are inconsistent. Moreover, whether the radiomics extracted from CECT images can provide Supplementary Information for differentiation of IAC from non-IAC remains unknown, especially for different types nodules (i.e., solid nodules, part-solid nodules, and pure ground-glass nodules).

Therefore, this study assessed the value of radiomics based on NCCT and CECT images to discriminate between IAC and non-IAC and compared the performances of models of different nodule subtypes (solid nodules, part-solid nodules and pure ground-glass nodules).

## Materials and methods

Our institutional review board approved this retrospective study (No. 2019K134) and waived the requirement of obtaining informed consent from patients.

### Study population

A total of 2,130 patients who underwent CECT examinations for pulmonary nodules between January 2014 and January 2019 were selected. Their medical records were reviewed for clinical characteristics, histopathological results, and serial chest CT scans. The inclusion criteria were as follows: (1) the presence of a pulmonary nodule; (2) histopathologically confirmed benign nodules, AAH, AIS, MIA, or IAC, or confirmed follow-up for inflammatory lesions; (3) NCCT and CECT scans were available and acquired sequentially in one examination; and (4) CT slice thickness ≤ 1.25 mm. The exclusion criteria were: (1) prior treatment before surgery; (2). poor quality CT images, and (3). lesions that were difficult to delineate clearly.

**FIGURE 1:**
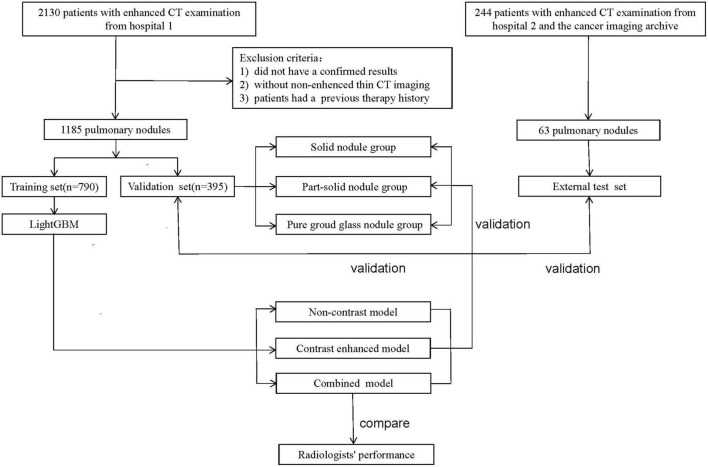
Workflow of the study.

Another 63 lung nodules met the inclusion and exclusion criteria were collected as external testing set to validate the stability and generalization of the models. Among the 63 nodules, 22 were selected from the cancer imaging archive (15) and 41 were collected from the Second Xiangya Hospital of Central South University. The workflow is described in Figure 1.

### Computed tomography scanning

Chest CT scanning was performed using one of following the four CT systems: GE Discovery CT750 HD, 64-slice LightSpeed VCT (both from GE Medical Systems), Somatom Definition Flash, and Somatom Sensation-16 (both from Siemens Medical Solutions). The detailed scan and reconstruction parameters are listed in Table 1. All patients received a bolus of 80–100 mL of intravenous contrast medium (Optiray; Mallinckrodt Imaging, MO, USA; 350 mg iodine per mL) at a rate of 3–4 mL/s using a power injector *via* an 18- or 20-gauge cannula into the antecubital vein. Enhanced CT scanning commenced 50–60 s after the administration of the contrast medium.

**TABLE 1:** Detailed scan and reconstruction parameters.

Setting	Tube voltage (kV)	Tube current (mA)	Pitch	Slice thickness of reconstruction (mm)	Slice interval of reconstruction (mm)	Reconstruction algorithm
GE Discovery CT750 HD	120	200	0.984:1	1.25	1.25	STND
Lightspeed VCT	120	200	0.984:1	1.25	1.25	STND
Somatom definition flash	120	110	1	1	1	Medium sharp
Somatom sensation-16	120	110	0.8	1	1	Medium sharp

### Pathological analysis

All resected specimens were formalin-fixed and stained with hematoxylin–eosin in accordance with the routine regulations of the hospital. A pathologist (with 10 years of experience in the pathological diagnosis of lung cancer) reviewed the specimens and recorded the pathological subtype of each nodule.

### Nodule labeling and segmentation

One radiologist with 5 years of experience in chest CT interpretation used a medical image processing and navigation software, 3D Slicer (version 4.8; National Institutes of Health)^1^, to manually delineate the volumes of interest of the 1,185 nodules at the voxel level in separate NCCT and CECT images. The volume of interest was confirmed by another radiologist with 12 years of experience in chest CT interpretation. DICOM images were imported into the software for delineation, and the label information was extracted with the nearly raw raster data format for further analysis. Each segmented nodule was given a specific label, non-IAC (inflammatory nodule, benign tumor, AAH, AIS, or MIA) or IAC. To assess the segmentation variability, a third radiologist with 3 years of experience in chest CT interpretation independently segmented a random set of 60 nodules to calculate the intra-class correlation coefficient (ICC) for each radiomic feature.

### Observer study

Two radiologists (a junior and a senior radiologist with more than 3 and 10 years of experience, respectively), who were blinded to the histopathological results and clinical data, independently classified and diagnosed all nodules in the validation set and external testing set. First, the two radiologists categorized the nodules as IAC or non-IAC based on the NCCT images, they then accessed to the folder containing the CECT images and diagnosed the nodules again using both the NCCT and CECT images.

### Extraction of radiomic features

Radiomic features were extracted using PyRadiomics 2.2.0^2^ (16), an open-source Python package for the extraction of radiomics. The process of extracting radiomic features is described in Supplementary material. To minimize the effect of image heterogeneity, we normalized the image spatial resolution and voxels before radiomic features extraction. A total of 1,218 features were extracted, including shape class, first-order class, gray level co-occurrence matrix (GLCM) class, gray level dependence matrix (GLDM) class, gray level size zone matrix (GLSM) class, and gray level run length matrix (GLRLM) class. We also used Min-Max scaling to normalize features before model construction. For feature variability analysis, the ICC for each radiomic feature was calculated using a two-way random-effects model under an absolute agreement condition. The reproducibility of the radiomic features was considered to be either high (ICC ≥ 0.8), intermediate (0.5 ≤ ICC < 0.8), or poor (ICC < 0.5). The radiomic features with high reproducibility were used as the input variables for building the diagnostic models.

### Building and validation of the diagnostic models

All patients were randomly assigned to a training set (*n* = 790) or a validation set (*n* = 395) at a ratio of 2:1 using the “scikit-learn” software packages for Python (17). The validation set was further divided into three subgroups, 91 solid nodules (Group 1), 239 part-solid nodules (Group 2), and 65 pure ground-glass nodules (Group 3). The distribution of different nodules properties (non-IAC vs. IAC, solid nodules vs. part-solid nodules vs. pure ground glass nodules) was kept uniform in both the training set and the validation set. After assessing the reproducibility based on the re-segmentation data, the open-source framework LightGBM was used for feature selection and model building in the training set (15). LightGBM is a fast, distributed, and efficient gradient boosting framework based on decision tree algorithms. Finally, the NCCT, CECT, and combined models differentiating between non-IAC and IAC were established. The performances of these models were then tested in the validation set (also in three subgroups) and external testing set.

### Statistical analysis

Differences in variables between the two patient groups were assessed using the independent-sample *t*-test or Mann–Whitney *U*-test for continuous variables and Fisher’s exact test or the chi-squared test for categorical variables. To assess the predictive performance of the study variables, receiver-operating characteristic (ROC) curves were plotted for the study variables to assess their predictive performance and compared using the DeLong test and the area under the curve (AUC) of the ROC curve was calculated. A two-sided *p*-value < 0.05 was considered statistically significant. Statistical analysis was performed using Python (Version 3.7.1) software and SPSS (Version 22.0, IBM).

## Results

### Patient profiles

A total of 1,185 nodules from 1,185 patients in our hospital were enrolled. Among the 1,185 patients, 690 were women (58.2%) and 495 were men (41.8%). The mean age of the patients was 58.95 ± 11.45 years (range: 20–81 years); the maximum diameter of the pulmonary nodules was 18.79 ± 11.32 mm (range: 5–82 mm). There were 478 (40.3%) nodules were diagnosed as non-IAC (123 inflammation or benign tumor; 11 AAH; 84 AIS; 260 MIA), and 707 (59.7%) IAC. Among the 1,185 nodules, 273 (23.0%) were solid nodules, 717 (60.5%) were part-solid nodules, and 195 (16.5%) were pure ground glass nodules. The patient information of the training set, validation set and external set are shown in Table 2. The patient information of the three subgroups are shown in Supplementary Table 1. Of the 63 pulmonary nodules in the external testing set, 22 were non-IAC and 41 were IAC. There were 28 (44.4%) solid nodules, 26 (41.3%) part-solid nodules and 9 (14.3) pure ground-glass nodules.

**TABLE 2:** Patient information of the training set, validation set and external set.

Demographic and clinical characteristic	Training set (*n* = 790)	Validation set (*n* = 395)	*p*	External validation set (*n* = 63)
Age (years)	58.89 ± 11.23	59.08 ± 11.83	0.789	60.05 ± 10.25
Size (mm)	18.56 ± 10.75	18.91 ± 10.53	0.598	22.8 ± 10.92
**Gender**			0.708	
Female	463 (58.6)	227 (57.5)		31 (49.2)
Male	327 (41.4)	168 (42.5)		32 (50.8)
**Pathology**			0.933	
IAC	474 (60.0)	233 (59.0)		41 (65.1)
**Non-IAC**				
Benign lesions	83 (10.5)	40 (10.1)		5 (7.9)
AAH	9 (1.1)	2 (0.5)		0
AIS	49 (6.2)	35 (8.9)		4 (6.3)
MIA	175 (22.2)	85 (21.5)		13 (20.6)
**Type**			1.000	
Pure ground glass nodule	130 (16.5)	91 (23.0)		9 (14.3)
Part-solid nodule	478 (60.5)	239 (60.5)		26 (41.3)
Solid nodule	182 (23.0)	65 (16.5)		28 (44.4)
**Location**			0.585	
Right upper lobe	274 (34.1)	135 (34.2)		20 (31.7)
Right middle lobe	64 (8.1)	39 (9.9)		3 (4.8)
Right lower lobe	153 (19.4)	78 (19.7)		10 (15.9)
Left lower lobe	191 (24.2)	99 (25.1)		17 (27.0)
Left lower lobe	108 (13.7)	44 (11.5)		13 (20.6)

IAC, invasive adenocarcinoma; AAH, atypical adenocarcinoma hyperplasia; AIS, adenocarcinoma *in situ*; MIA, minimally invasive adenocarcinoma.

### Model building and diagnostic validation

After reproducibility analysis, 534 features on NCCT and 559 features on CECT remained separate (ICCs ≥ 0.8), and the details are shown in Supplementary Tables 2, 3. The selected features were inputted into the LightGBM framework to construct the NCCT, CECT and combined models. LightGBM ranked the importance of features based on the number of times they were used in the decision tree.

**FIGURE 2:**
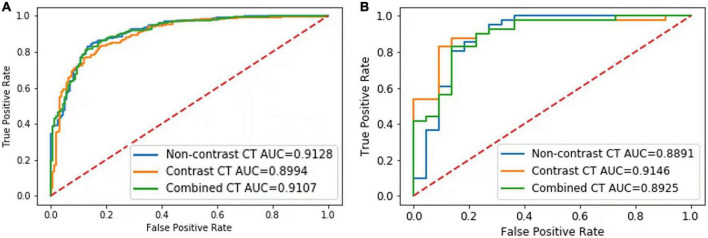
Results of the receiver-operating characteristic (ROC) curve analysis. The ROC curves of the NCCT, CECT, and combined models for identification of invasive adenocarcinoma (IAC) in the validation set **(A)** and external testing set **(B)** are shown.

In the validation set, the AUCs of the NCCT, CECT, and combined models were 0.91, 0.90 and 0.91 respectively, to distinguish IAC and non-IAC cases (Figure 2A). The DeLong test found no statistically significant difference among the three models (NCCT model vs. CECT model, *P* = 0.247; NCCT model vs. combined model, *P* = 0.320; CECT model vs. combined model, *P* = 0.277). In the external testing set, the AUCs of the NCCT, CECT, and combined models were 0.89, 0.91, and 0.89, respectively (Figure 2B). Again, no statistically significant differences among the three models were identified by the DeLong test (NCCT model vs. CECT model, *P* = 0.218; NCCT model vs. combined model, *P* = 0.436; and CECT model vs. combined model, *P* = 0.148). The accuracies of the radiomics models were close to those of the senior radiologist and better than those of the junior radiologist for both the validation set and external testing set (Table 3).

**TABLE 3:** Performance of the radiomics models and radiologists for lung IAC.

		Radiomics models			Junior radiologist		Senior radiologist	
		NCCT	CECT	NCCT + CECT	NCCT	NCCT + CECT	NCCT	NCCT + CECT
Validation set	Accuracy	82.74%	81.47%	83.50%	74.90%	76.90%	83.40%	83.90%
	F1	0.86	0.95	0.87				
	AUC	0.91	0.90	0.91				
Group 1	Accuracy	74.73%	68.13%	74.73%	66.70%	68.90%	80.00%	84.40%
	F1	0.82	0.79	0.82				
	AUC	0.82	0.79	0.81				
Group 2	Accuracy	85.71%	86.55%	86.55%	76.30%	77.50%	83.50%	82.60%
	F1	0.90	0.90	0.90				
	AUC	0.93	0.92	0.93				
Group 3	Accuracy	83.08%	81.54%	84.62%	81.50%	82.20%	87.70%	87.70%
	F1	0.86	0.84	0.88				
	AUC	0.90	0.90	0.89				
External testing set	Accuracy	84.13%	84.13%	84.13%	75.34%	76.12%	84.45%	85.21%
	F1	0.88	0.88	0.88				
	AUC	0.89	0.91	0.89				

### Performance of the models in the subgroups

**FIGURE 3:**
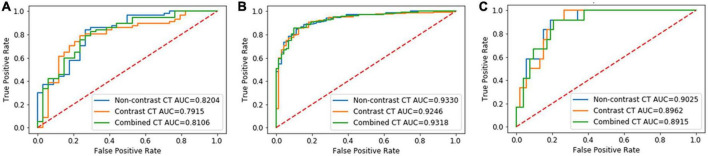
Results of the receiver-operating characteristic (ROC) curve analysis. The ROC curves of the NCCT, CECT, and combined models for identification of invasive adenocarcinoma (IAC) in solid nodule group **(A)**, partly solid nodule group **(B)**, and pure ground glass nodule group **(C)** are shown.

In Group 1, the AUCs of the NCCT, CECT, and combined models were 0.82, 0.79, and 0.81, respectively, without significant difference in the DeLong test (NCCT model vs. CECT model, *P* = 0.247; NCCT model vs. combined model, *P* = 0.320; and CECT model vs. combined model, *P* = 0.277) (Figure 3A). The accuracies of the radiomics models were slightly better than that of the junior radiologist but significantly lower than that of the senior radiologist (Table 3). In Group 2, the AUCs of the NCCT, CECT, and combined model were 0.93, 0.92, and 0.93, respectively (Figure 3B). The results of the DeLong test showed no statistically significant differences among the three models (NCCT vs. CECT model, *P* = 0.159; NCCT vs. combined model, *P* = 0.402; and CECT vs. combined model, *P* = 0.160). In this group, the accuracies of the radiomics models were better than those of the junior and senior radiologists (Table 3). In Group 3, the AUCs of the NCCT, CECT, and combined model in Group 3 were 0.90, 0.90, and 0.89, respectively (Figure 3C). The DeLong test showed no statistically significant differences among the three models (NCCT vs. CECT model, *P* = 0.402; NCCT vs. combined model, *P* = 0.213; and CECT vs. combined model, *P* = 0.406). The accuracies of the radiomics models were close to that of the junior radiologist but lower than that of the senior radiologist (Table 3).

### Top 10 features of the non-contrast computed tomography, contrast-enhanced computed tomography, and combined models

**FIGURE 4:**
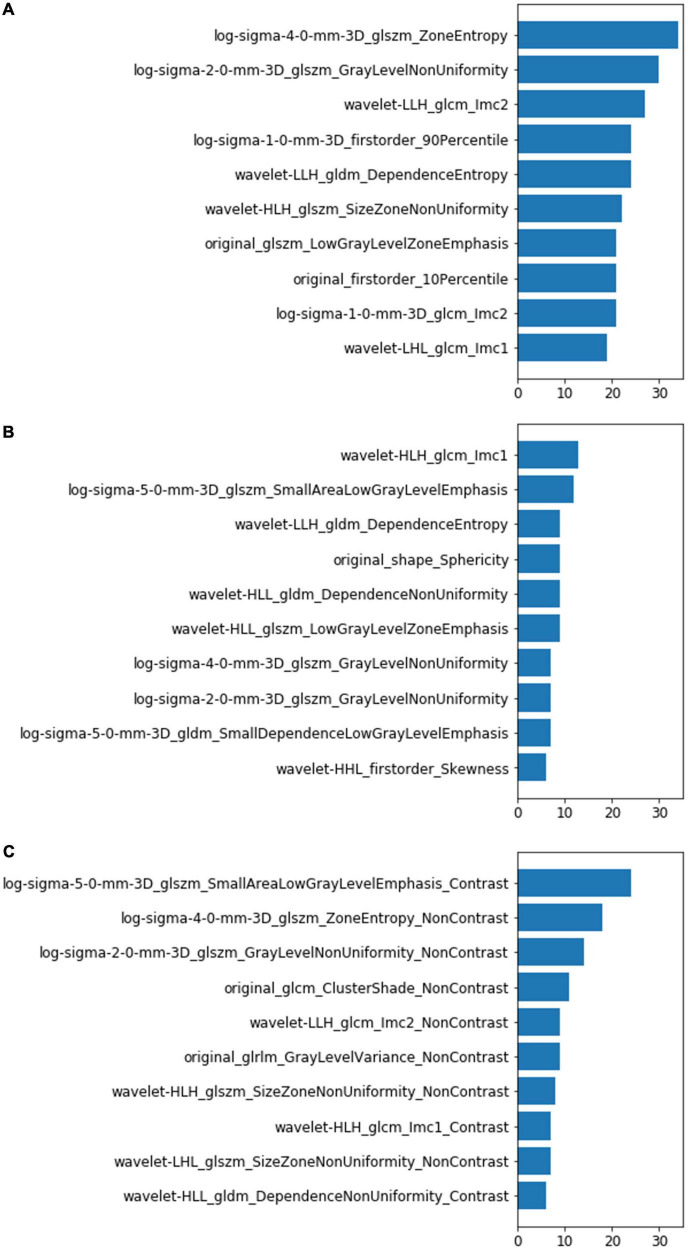
Top 10 most-used features of the NCCT model **(A)**, CECT model **(B)**, and combined model **(C)**. The left vertical coordinates indicate the radiomic features; the horizontal coordinates indicate the number of times the features were used in the models.

The LightGBM framework ranked the importance of features according to the number of times they were used in the decision tree. The top 10 features of the models were listed in Figure 4. Most of the features were different, and only one feature (wavelet_gldm_DependenceEntropy) was same between the top 10 features of the NCCN model and CECT model. Seven of the combined model’s top 10 features were from NCCT images and three features were from CECT images. Only six of the combined model’s top 10 features appeared in the NCCT and CECT models. Of all three models’ top 10 features, thirteen were from GLSZM, seven were from GLCM, five were from GLDM, three were from first-order, one was from shape and one was from GLRLM separately. Of the thirteen features from GLSZM, four were in the NCCT model, four were in the NCCT model and five were in the combined model.

## Discussion

This study investigated the value of radiomic features extracted from NCCT and CECT images in the diagnosis of IAC/non-IAC. The radiomics models showed good predictive performance in discriminating between IAC and non-IAC of the lung, especially those with in part-solid nodules. Generally, the accuracies of the radiomic models were close to that of the senior radiologist and better than that of the junior radiologist. However, the radiomic models based on CECT images provided no additional value compared to the NCCT models.

To date, several studies have documented that CT-based radiomics can identify lung IAC with AUCs of 0.77–0.90 (18). Our NCCT model also obtained good performance (AUC = 0.91 in validation set), verifying the ability of CT based radiomics for identifying IAC. However, previous radiomics studies were rarely based on CECT images. Recently, radiomics extracted from CECT images were investigated, however, the results were inconsistent. Chen et al. demonstrated that the radiomics model based on CECT could provide additional value in the prediction of invasiveness of subcentimeter ground glass nodules (AUC_CECT: 0.896 vs. AUC_NCCT: 0.851) (19). In the study of Fan et al., a radiomics model was constructed using NCCT images for IAC prediction showed similar performance in NCCT validation set and CECT validation set (7). This result suggests that contrast injection did not affect the two features included in their radiomics model (i.e., GLCM_correlation and GLCM_cluster_tendency). Other studies also constructed radiomics models separately based on NCCT and CECT images and compared their performance for predicting lung IAC. Gao et al. enrolled 34 IACs that appeared as ground glass nodules and constructed models using multivariate logistic regression analysis (14). Their results also suggested that CECT did not improve the performance of the radiomics model. For solid nodules, Yang et al. (18) constructed radiomics models for differentiating granulomatous nodules from lung adenocarcinoma; they came to the same conclusion. Our result showed that the NCCT, CECT, and combined model achieved similar performance for identifying lung IAC. In subgroups, the AUCs of the three models also showed no statistically significant difference. Our study enrolled pure ground-glass nodules, partly solid nodules, and solid nodules, built models that merged the three types of nodules, and validated them in three subgroups. To minimize interference factors caused by multiple scans (such as CT scanners and protocols), we excluded nodules whose NCCT and CECT images were not acquired in one examination. Our results suggested that CECT did not improve the radiomics performance for lung IAC prediction either in solid nodules or ground glass nodules. We considered the possible reasons were: (1) The existence of contrast agents within the tumor may reduce the biological heterogeneity that facilitates the differentiation between benign and malignant nodules. (2) Calibration before model building might reduce the image intensity.

In subgroup analysis, although there was no statistically difference between the AUCs of the radiomics models within the groups, there was a significant difference between groups. The performances of the models were significantly lower in solid nodule group than those in the part-solid nodule group and pure ground-glass nodule group. This result is with that of other studies, although they only included one type of nodules. While Wu et al. (20) showed that a radiomics model for the prediction of lung IAC (part solid nodules) obtained an AUC of 0.88., Yang et al. (18) reported that radiomics models for differentiating solitary granulomatous with solid IAC achieved low AUCs (AUC_NNCT = 0.78, AUC_CECT = 0.77, and AUC_combined = 0.80). In another study (21), a radiomics model achieved an AUC of 0.967 for differentiating solid lung adenocarcinoma from benign lesions; this is obviously better than our result. A possible reason for this is that solid nodules only represented only a small percentage of our training set, and the model cannot generate diagnostic information. In addition, we found that the accuracies of the radiomics models were both superior to those of the junior radiologist and senior radiologist for the part solid nodule group. The solid components in nodules, which are crucial for identifying IAC (GGN) with ground glass nodules, are diverse pathologically and include mucus, hemorrhage, mucus, granulation tissue, and alveolar collapse. It is rather difficult for radiologists to differentiate these solid components in many cases, but some invisible radiomic feature may reflect their differences.

Although the performances of the NCCT and CECT models were similar, the top features they used differed greatly. This suggests that the contrast agent changed many radiomic features and affected their predictive power. In the combined model, more features were from NCCT(7/10)than from CECT (3/10); this phenomena may explain why CECT did not improve the model performance. Among the three model’ top 10 features, 13/30 were from GLSZM class (4 in NCCT model, 4 in CECT model and 5 in combined model). GLSZM quantifies gray level zones in an image, which is defined as the number of connected voxels that share the same gray level intensity. This may indicate that GLSZM features are more stable and critical for lung IAC prediction.

This study has several limitations. First, it was limited by its retrospective nature. The heterogeneity of imaging protocols and image quality may have affected the result. Second, we did not validate the performance of models in subgroups of external set due to the limited data; therefore the subgroup results need to be confirmed. Third, the malignant group comprised only adenocarcinoma; thus, the results of this study cannot address the situation in other pulmonary malignant tumors.

In conclusion, the CT image based radiomics models showed good predictive performance in the diagnosis of lung invasive adenocarcinoma, especially those with part solid nodules; however, the radiomic model based on CECT images provided no additional value. In the diagnosis of pulmonary nodules, enhanced CT examinations should be selected cautiously, especially in young patients and patients with impaired renal function.

## Data availability statement

The raw data supporting the conclusions of this article will be made available by the authors, without undue reservation.

## Ethics statement

Our Institutional Review Board approved this Retrospective Study (no. 2019K134) and waived the requirement of obtaining informed consent from patients. Written informed consent for participation was not required for this study in accordance with the national legislation and the institutional requirements.

## Author contributions

YS and WZ: conceptualization, methodology, writing original draft preparation, and investigation. LJ, PG, YX, and JL: data curation and visualization. KK and SD: software and validation. ML and JL: writing—reviewing, supervision, and editing. All authors contributed to the article and approved the submitted version.

## Footnotes

1
https://www.slicer.org

2
https://pyradiomics.readthedocs.io/en/latest/index.html
