# Supplementary material for: Non-contrast and contrast enhanced computed tomography radiomics in preoperative discrimination of lung invasive and non-invasive adenocarcinoma
Source: Front Med (Lausanne). 2022 Nov 4;9:939434. doi: 10.3389/fmed.2022.939434 (PMC9672504; doi:10.3389/fmed.2022.939434)
Supplement: Supplementary file 1 [file Data_Sheet_1.DOCX]

**Supplementary** **Methods**

**Radiomics features extraction methodology**

Radiomic features were extracted using PyRadiomics 2.2.0 (https://pyradiomics.readthedocs.io/en/latest/index.html), an open-source python package for the extraction of radiomics. After image normalization and segmentation, NCCT and CECT images (NIfTI format) and the corresponding volume of interest (VOI) file (NIfTI format)were inputted to pyradiomics software. At initialization, a parameters file (exampleCT.yaml)containing all necessary settings (top level containing keys “setting”, “imageType” and/or “featureClass) was used. The parameters file was provided in the exampleSettings folder in the above website.

**Supplement Tables**

**TABLE 1.** Patient Information of the Three Subgroups.

| Demographic and Clinical Characteristic | | Group 1  (solid nodules, N=91) | Group 2 (part-solid nodules,  N=239) | Group 3 (pure ground-glass nodules, N=65) |
| --- | --- | --- | --- | --- |
| Age(years) | | 56.70±11.22 | 59.83±11.94 | 55.45±11.75 |
| Size(mm) | | 22.60±10.80 | 19.62±10.61 | 13.09±17.59 |
| Gender | |  |  |  |
|  | Male | 38(41.8) | 94(39.3) | 21(32.3) |
|  | Female | 53(58.2) | 145(60.7) | 44(67.7) |
| pathology | |  |  |  |
|  | IAC | 57(62.6) | 166(69.5) | 12(18.5) |
|  | non-IAC | 34(37.4) | 73(30.5) | 53(81.5) |
| Location | |  |  |  |
|  | Right upper lobe | 25(27.5) | 83(34.7) | 27(41.5) |
|  | Right middle lobe | 11(12.1) | 19(7.9) | 9(13.8) |
|  | Right lower lobe | 20(22.0) | 50(20.9) | 8(12.3) |
|  | Left lower lobe | 23(25.3) | 61(25.5) | 15(23.1) |
|  | Left lower lobe | 12(13.2) | 26(10.9) | 6(9.2) |

**TABLE 2:** Radiomics Features with ICC≥0.8 in Non-contrast Enhanced CT Image.

| **Radiomic Feature** | **ICC value** |
| --- | --- |
| original_shape_Flatness | 0.835 |
| original_shape_LeastAxisLength | 0.928 |
| original_shape_MajorAxisLength | 0.928 |
| original_shape_Maximum2DDiameterRow | 0.883 |
| original_shape_Maximum2DDiameterSlice | 0.956 |
| original_shape_Sphericity | 0.968 |
| original_shape_SurfaceArea | 0.910 |
| original_shape_SurfaceVolumeRatio | 0.835 |
| original_firstorder_10Percentile | 0.847 |
| original_firstorder_90Percentile | 0.972 |
| original_firstorder_Energy | 0.895 |
| original_firstorder_Kurtosis | 0.862 |
| original_firstorder_Maximum | 0.951 |
| original_firstorder_MeanAbsoluteDeviation | 0.972 |
| original_firstorder_Median | 0.923 |
| original_firstorder_Minimum | 0.959 |
| original_firstorder_RootMeanSquared | 0.951 |
| original_firstorder_Skewness | 0.912 |
| original_firstorder_TotalEnergy | 0.895 |
| original_firstorder_Variance | 0.968 |
| original_glcm_Autocorrelation | 0.962 |
| original_glcm_ClusterShade | 0.975 |
| original_glcm_ClusterTendency | 0.950 |
| original_glcm_Contrast | 0.941 |
| original_glcm_DifferenceAverage | 0.942 |
| original_glcm_DifferenceEntropy | 0.980 |
| original_glcm_JointEntropy | 0.983 |
| original_glcm_Imc2 | 0.952 |
| original_glcm_Idm | 0.951 |
| original_glcm_InverseVariance | 0.956 |
| original_glcm_MaximumProbability | 0.965 |
| original_glrlm_GrayLevelNonUniformityNormalized | 0.990 |
| original_glrlm_GrayLevelVariance | 0.969 |
| original_glrlm_HighGrayLevelRunEmphasis | 0.963 |
| original_glrlm_LongRunHighGrayLevelEmphasis | 0.971 |
| original_glrlm_LongRunLowGrayLevelEmphasis | 0.985 |
| original_glrlm_RunLengthNonUniformityNormalized | 0.930 |
| original_glrlm_RunPercentage | 0.936 |
| original_glrlm_RunVariance | 0.952 |
| original_glrlm_ShortRunHighGrayLevelEmphasis | 0.962 |
| original_glrlm_ShortRunLowGrayLevelEmphasis | 0.991 |
| original_glszm_HighGrayLevelZoneEmphasis | 0.971 |
| original_glszm_LargeAreaEmphasis | 0.974 |
| original_glszm_LargeAreaHighGrayLevelEmphasis | 1.000 |
| original_glszm_LowGrayLevelZoneEmphasis | 0.991 |
| original_glszm_SizeZoneNonUniformity | 0.952 |
| original_glszm_SmallAreaHighGrayLevelEmphasis | 0.968 |
| original_glszm_SmallAreaLowGrayLevelEmphasis | 0.956 |
| original_glszm_ZoneEntropy | 0.973 |
| original_glszm_ZoneVariance | 0.974 |
| original_gldm_DependenceEntropy | 0.941 |
| original_gldm_GrayLevelVariance | 0.968 |
| original_gldm_HighGrayLevelEmphasis | 0.963 |
| original_gldm_LargeDependenceEmphasis | 0.956 |
| original_gldm_LargeDependenceLowGrayLevelEmphasis | 0.948 |
| original_gldm_LowGrayLevelEmphasis | 0.988 |
| original_gldm_SmallDependenceLowGrayLevelEmphasis | 0.940 |
| log-sigma-1-0-mm-3D_firstorder_10Percentile | 0.919 |
| log-sigma-1-0-mm-3D_firstorder_90Percentile | 0.857 |
| log-sigma-1-0-mm-3D_firstorder_InterquartileRange | 0.948 |
| log-sigma-1-0-mm-3D_firstorder_Kurtosis | 0.859 |
| log-sigma-1-0-mm-3D_firstorder_Minimum | 0.976 |
| log-sigma-1-0-mm-3D_firstorder_Range | 0.993 |
| log-sigma-1-0-mm-3D_firstorder_RobustMeanAbsoluteDeviation | 0.963 |
| log-sigma-1-0-mm-3D_firstorder_Uniformity | 0.984 |
| log-sigma-1-0-mm-3D_firstorder_Variance | 0.975 |
| log-sigma-1-0-mm-3D_glcm_ClusterProminence | 0.966 |
| log-sigma-1-0-mm-3D_glcm_ClusterTendency | 0.985 |
| log-sigma-1-0-mm-3D_glcm_Contrast | 0.944 |
| log-sigma-1-0-mm-3D_glcm_DifferenceAverage | 0.948 |
| log-sigma-1-0-mm-3D_glcm_DifferenceEntropy | 0.979 |
| log-sigma-1-0-mm-3D_glcm_JointEntropy | 0.990 |
| log-sigma-1-0-mm-3D_glcm_Imc2 | 0.938 |
| log-sigma-1-0-mm-3D_glcm_Idm | 0.945 |
| log-sigma-1-0-mm-3D_glcm_Id | 0.945 |
| log-sigma-1-0-mm-3D_glcm_Idn | 0.877 |
| log-sigma-1-0-mm-3D_glcm_SumEntropy | 0.992 |
| log-sigma-1-0-mm-3D_glcm_SumSquares | 0.977 |
| log-sigma-1-0-mm-3D_glrlm_GrayLevelNonUniformityNormalized | 0.986 |
| log-sigma-1-0-mm-3D_glrlm_HighGrayLevelRunEmphasis | 0.960 |
| log-sigma-1-0-mm-3D_glrlm_LongRunEmphasis | 0.941 |
| log-sigma-1-0-mm-3D_glrlm_LowGrayLevelRunEmphasis | 0.995 |
| log-sigma-1-0-mm-3D_glrlm_RunEntropy | 0.995 |
| log-sigma-1-0-mm-3D_glrlm_RunLengthNonUniformityNormalized | 0.912 |
| log-sigma-1-0-mm-3D_glrlm_RunVariance | 0.939 |
| log-sigma-1-0-mm-3D_glrlm_ShortRunEmphasis | 0.926 |
| log-sigma-1-0-mm-3D_glszm_GrayLevelNonUniformityNormalized | 0.994 |
| log-sigma-1-0-mm-3D_glszm_GrayLevelVariance | 0.991 |
| log-sigma-1-0-mm-3D_glszm_HighGrayLevelZoneEmphasis | 0.978 |
| log-sigma-1-0-mm-3D_glszm_LargeAreaHighGrayLevelEmphasis | 0.936 |
| log-sigma-1-0-mm-3D_glszm_LargeAreaLowGrayLevelEmphasis | 0.990 |
| log-sigma-1-0-mm-3D_glszm_SmallAreaEmphasis | 0.843 |
| log-sigma-1-0-mm-3D_glszm_SmallAreaHighGrayLevelEmphasis | 0.984 |
| log-sigma-1-0-mm-3D_glszm_ZoneEntropy | 0.982 |
| log-sigma-1-0-mm-3D_glszm_ZoneVariance | 0.995 |
| log-sigma-1-0-mm-3D_gldm_DependenceEntropy | 0.947 |
| log-sigma-1-0-mm-3D_gldm_HighGrayLevelEmphasis | 0.958 |
| log-sigma-1-0-mm-3D_gldm_LargeDependenceEmphasis | 0.943 |
| log-sigma-1-0-mm-3D_gldm_LargeDependenceHighGrayLevelEmphasis | 0.816 |
| log-sigma-1-0-mm-3D_gldm_LowGrayLevelEmphasis | 0.996 |
| log-sigma-1-0-mm-3D_gldm_SmallDependenceEmphasis | 0.884 |
| log-sigma-2-0-mm-3D_firstorder_10Percentile | 0.964 |
| log-sigma-2-0-mm-3D_firstorder_Entropy | 0.995 |
| log-sigma-2-0-mm-3D_firstorder_InterquartileRange | 0.982 |
| log-sigma-2-0-mm-3D_firstorder_Maximum | 0.938 |
| log-sigma-2-0-mm-3D_firstorder_MeanAbsoluteDeviation | 0.992 |
| log-sigma-2-0-mm-3D_firstorder_Range | 0.975 |
| log-sigma-2-0-mm-3D_firstorder_RobustMeanAbsoluteDeviation | 0.988 |
| log-sigma-2-0-mm-3D_firstorder_RootMeanSquared | 0.825 |
| log-sigma-2-0-mm-3D_firstorder_Uniformity | 0.993 |
| log-sigma-2-0-mm-3D_firstorder_Variance | 0.985 |
| log-sigma-2-0-mm-3D_glcm_ClusterProminence | 0.862 |
| log-sigma-2-0-mm-3D_glcm_ClusterShade | 0.861 |
| log-sigma-2-0-mm-3D_glcm_ClusterTendency | 0.970 |
| log-sigma-2-0-mm-3D_glcm_Correlation | 0.896 |
| log-sigma-2-0-mm-3D_glcm_DifferenceAverage | 0.969 |
| log-sigma-2-0-mm-3D_glcm_JointEnergy | 0.990 |
| log-sigma-2-0-mm-3D_glcm_JointEntropy | 0.994 |
| log-sigma-2-0-mm-3D_glcm_Imc1 | 0.883 |
| log-sigma-2-0-mm-3D_glcm_Idm | 0.953 |
| log-sigma-2-0-mm-3D_glcm_Idmn | 0.882 |
| log-sigma-2-0-mm-3D_glcm_InverseVariance | 0.953 |
| log-sigma-2-0-mm-3D_glcm_MaximumProbability | 0.961 |
| log-sigma-2-0-mm-3D_glcm_SumEntropy | 0.995 |
| log-sigma-2-0-mm-3D_glrlm_GrayLevelNonUniformityNormalized | 0.996 |
| log-sigma-2-0-mm-3D_glrlm_GrayLevelVariance | 0.982 |
| log-sigma-2-0-mm-3D_glrlm_RunEntropy | 0.982 |
| log-sigma-2-0-mm-3D_glrlm_RunLengthNonUniformityNormalized | 0.921 |
| log-sigma-2-0-mm-3D_glrlm_RunPercentage | 0.919 |
| log-sigma-2-0-mm-3D_glrlm_ShortRunEmphasis | 0.935 |
| log-sigma-2-0-mm-3D_glrlm_ShortRunHighGrayLevelEmphasis | 0.918 |
| log-sigma-2-0-mm-3D_glszm_GrayLevelNonUniformity | 0.831 |
| log-sigma-2-0-mm-3D_glszm_GrayLevelNonUniformityNormalized | 0.996 |
| log-sigma-2-0-mm-3D_glszm_GrayLevelVariance | 0.940 |
| log-sigma-2-0-mm-3D_glszm_HighGrayLevelZoneEmphasis | 0.947 |
| log-sigma-2-0-mm-3D_glszm_LargeAreaHighGrayLevelEmphasis | 0.865 |
| log-sigma-2-0-mm-3D_glszm_LowGrayLevelZoneEmphasis | 0.963 |
| log-sigma-2-0-mm-3D_glszm_SmallAreaEmphasis | 0.857 |
| log-sigma-2-0-mm-3D_glszm_SmallAreaHighGrayLevelEmphasis | 0.974 |
| log-sigma-2-0-mm-3D_glszm_SmallAreaLowGrayLevelEmphasis | 0.950 |
| log-sigma-2-0-mm-3D_glszm_ZonePercentage | 0.896 |
| log-sigma-2-0-mm-3D_glszm_ZoneVariance | 0.990 |
| log-sigma-2-0-mm-3D_gldm_HighGrayLevelEmphasis | 0.885 |
| log-sigma-2-0-mm-3D_gldm_LargeDependenceEmphasis | 0.919 |
| log-sigma-2-0-mm-3D_gldm_SmallDependenceEmphasis | 0.926 |
| log-sigma-2-0-mm-3D_gldm_SmallDependenceLowGrayLevelEmphasis | 0.804 |
| log-sigma-3-0-mm-3D_firstorder_10Percentile | 0.985 |
| log-sigma-3-0-mm-3D_firstorder_InterquartileRange | 0.993 |
| log-sigma-3-0-mm-3D_firstorder_Kurtosis | 0.832 |
| log-sigma-3-0-mm-3D_firstorder_Maximum | 0.941 |
| log-sigma-3-0-mm-3D_firstorder_Mean | 0.934 |
| log-sigma-3-0-mm-3D_firstorder_Median | 0.878 |
| log-sigma-3-0-mm-3D_firstorder_RobustMeanAbsoluteDeviation | 0.993 |
| log-sigma-3-0-mm-3D_firstorder_RootMeanSquared | 0.927 |
| log-sigma-3-0-mm-3D_firstorder_Skewness | 0.803 |
| log-sigma-3-0-mm-3D_firstorder_Variance | 0.993 |
| log-sigma-3-0-mm-3D_glcm_Autocorrelation | 0.928 |
| log-sigma-3-0-mm-3D_glcm_ClusterShade | 0.878 |
| log-sigma-3-0-mm-3D_glcm_ClusterTendency | 0.985 |
| log-sigma-3-0-mm-3D_glcm_Contrast | 0.992 |
| log-sigma-3-0-mm-3D_glcm_DifferenceAverage | 0.985 |
| log-sigma-3-0-mm-3D_glcm_DifferenceEntropy | 0.995 |
| log-sigma-3-0-mm-3D_glcm_JointEntropy | 0.996 |
| log-sigma-3-0-mm-3D_glcm_Imc1 | 0.839 |
| log-sigma-3-0-mm-3D_glcm_Imc2 | 0.917 |
| log-sigma-3-0-mm-3D_glcm_Idmn | 0.839 |
| log-sigma-3-0-mm-3D_glcm_Id | 0.972 |
| log-sigma-3-0-mm-3D_glcm_MaximumProbability | 0.931 |
| log-sigma-3-0-mm-3D_glcm_SumEntropy | 0.996 |
| log-sigma-3-0-mm-3D_glcm_SumSquares | 0.987 |
| log-sigma-3-0-mm-3D_glrlm_GrayLevelVariance | 0.991 |
| log-sigma-3-0-mm-3D_glrlm_HighGrayLevelRunEmphasis | 0.953 |
| log-sigma-3-0-mm-3D_glrlm_LowGrayLevelRunEmphasis | 0.964 |
| log-sigma-3-0-mm-3D_glrlm_RunEntropy | 0.980 |
| log-sigma-3-0-mm-3D_glrlm_RunLengthNonUniformityNormalized | 0.958 |
| log-sigma-3-0-mm-3D_glrlm_RunVariance | 0.907 |
| log-sigma-3-0-mm-3D_glrlm_ShortRunEmphasis | 0.969 |
| log-sigma-3-0-mm-3D_glszm_GrayLevelNonUniformity | 0.985 |
| log-sigma-3-0-mm-3D_glszm_GrayLevelNonUniformityNormalized | 0.832 |
| log-sigma-3-0-mm-3D_glszm_GrayLevelVariance | 0.953 |
| log-sigma-3-0-mm-3D_glszm_LargeAreaLowGrayLevelEmphasis | 0.874 |
| log-sigma-3-0-mm-3D_glszm_LowGrayLevelZoneEmphasis | 0.994 |
| log-sigma-3-0-mm-3D_glszm_SmallAreaEmphasis | 0.935 |
| log-sigma-3-0-mm-3D_glszm_SmallAreaHighGrayLevelEmphasis | 0.993 |
| log-sigma-3-0-mm-3D_glszm_SmallAreaLowGrayLevelEmphasis | 0.852 |
| log-sigma-3-0-mm-3D_glszm_ZonePercentage | 0.941 |
| log-sigma-3-0-mm-3D_gldm_DependenceEntropy | 0.948 |
| log-sigma-3-0-mm-3D_gldm_HighGrayLevelEmphasis | 0.947 |
| log-sigma-3-0-mm-3D_gldm_LargeDependenceEmphasis | 0.934 |
| log-sigma-3-0-mm-3D_gldm_LargeDependenceLowGrayLevelEmphasis | 0.953 |
| log-sigma-3-0-mm-3D_gldm_SmallDependenceEmphasis | 0.962 |
| log-sigma-3-0-mm-3D_gldm_SmallDependenceHighGrayLevelEmphasis | 0.986 |
| log-sigma-4-0-mm-3D_firstorder_90Percentile | 0.953 |
| log-sigma-4-0-mm-3D_firstorder_Entropy | 0.996 |
| log-sigma-4-0-mm-3D_firstorder_InterquartileRange | 0.990 |
| log-sigma-4-0-mm-3D_firstorder_MeanAbsoluteDeviation | 0.992 |
| log-sigma-4-0-mm-3D_firstorder_Mean | 0.972 |
| log-sigma-4-0-mm-3D_firstorder_Range | 0.990 |
| log-sigma-4-0-mm-3D_firstorder_RobustMeanAbsoluteDeviation | 0.991 |
| log-sigma-4-0-mm-3D_firstorder_RootMeanSquared | 0.969 |
| log-sigma-4-0-mm-3D_firstorder_Variance | 0.993 |
| log-sigma-4-0-mm-3D_glcm_Autocorrelation | 0.968 |
| log-sigma-4-0-mm-3D_glcm_ClusterShade | 0.878 |
| log-sigma-4-0-mm-3D_glcm_ClusterTendency | 0.989 |
| log-sigma-4-0-mm-3D_glcm_Contrast | 0.995 |
| log-sigma-4-0-mm-3D_glcm_DifferenceAverage | 0.992 |
| log-sigma-4-0-mm-3D_glcm_DifferenceEntropy | 0.996 |
| log-sigma-4-0-mm-3D_glcm_JointEntropy | 0.997 |
| log-sigma-4-0-mm-3D_glcm_Imc1 | 0.835 |
| log-sigma-4-0-mm-3D_glcm_Imc2 | 0.943 |
| log-sigma-4-0-mm-3D_glcm_Idmn | 0.981 |
| log-sigma-4-0-mm-3D_glcm_Id | 0.987 |
| log-sigma-4-0-mm-3D_glcm_MaximumProbability | 0.941 |
| log-sigma-4-0-mm-3D_glcm_SumEntropy | 0.995 |
| log-sigma-4-0-mm-3D_glcm_SumSquares | 0.990 |
| log-sigma-4-0-mm-3D_glrlm_GrayLevelVariance | 0.993 |
| log-sigma-4-0-mm-3D_glrlm_HighGrayLevelRunEmphasis | 0.978 |
| log-sigma-4-0-mm-3D_glrlm_LongRunLowGrayLevelEmphasis | 0.902 |
| log-sigma-4-0-mm-3D_glrlm_LowGrayLevelRunEmphasis | 0.983 |
| log-sigma-4-0-mm-3D_glrlm_RunEntropy | 0.979 |
| log-sigma-4-0-mm-3D_glrlm_RunPercentage | 0.960 |
| log-sigma-4-0-mm-3D_glrlm_RunVariance | 0.890 |
| log-sigma-4-0-mm-3D_glrlm_ShortRunLowGrayLevelEmphasis | 0.991 |
| log-sigma-4-0-mm-3D_glszm_GrayLevelNonUniformity | 0.929 |
| log-sigma-4-0-mm-3D_glszm_GrayLevelNonUniformityNormalized | 0.951 |
| log-sigma-4-0-mm-3D_glszm_HighGrayLevelZoneEmphasis | 0.980 |
| log-sigma-4-0-mm-3D_glszm_LowGrayLevelZoneEmphasis | 0.999 |
| log-sigma-4-0-mm-3D_glszm_SmallAreaEmphasis | 0.834 |
| log-sigma-4-0-mm-3D_glszm_SmallAreaHighGrayLevelEmphasis | 0.960 |
| log-sigma-4-0-mm-3D_glszm_SmallAreaLowGrayLevelEmphasis | 0.990 |
| log-sigma-4-0-mm-3D_glszm_ZoneEntropy | 0.991 |
| log-sigma-4-0-mm-3D_glszm_ZonePercentage | 0.929 |
| log-sigma-4-0-mm-3D_gldm_DependenceEntropy | 0.947 |
| log-sigma-4-0-mm-3D_gldm_HighGrayLevelEmphasis | 0.976 |
| log-sigma-4-0-mm-3D_gldm_LargeDependenceEmphasis | 0.956 |
| log-sigma-4-0-mm-3D_gldm_LargeDependenceLowGrayLevelEmphasis | 0.877 |
| log-sigma-4-0-mm-3D_gldm_SmallDependenceEmphasis | 0.975 |
| log-sigma-4-0-mm-3D_gldm_SmallDependenceHighGrayLevelEmphasis | 0.989 |
| log-sigma-5-0-mm-3D_firstorder_90Percentile | 0.951 |
| log-sigma-5-0-mm-3D_firstorder_Entropy | 0.991 |
| log-sigma-5-0-mm-3D_firstorder_InterquartileRange | 0.991 |
| log-sigma-5-0-mm-3D_firstorder_Maximum | 0.969 |
| log-sigma-5-0-mm-3D_firstorder_MeanAbsoluteDeviation | 0.992 |
| log-sigma-5-0-mm-3D_firstorder_Minimum | 0.999 |
| log-sigma-5-0-mm-3D_firstorder_Range | 0.993 |
| log-sigma-5-0-mm-3D_firstorder_RobustMeanAbsoluteDeviation | 0.992 |
| log-sigma-5-0-mm-3D_firstorder_Uniformity | 0.966 |
| log-sigma-5-0-mm-3D_firstorder_Variance | 0.989 |
| log-sigma-5-0-mm-3D_glcm_ClusterProminence | 0.975 |
| log-sigma-5-0-mm-3D_glcm_ClusterTendency | 0.993 |
| log-sigma-5-0-mm-3D_glcm_Contrast | 0.999 |
| log-sigma-5-0-mm-3D_glcm_DifferenceAverage | 0.997 |
| log-sigma-5-0-mm-3D_glcm_DifferenceEntropy | 0.991 |
| log-sigma-5-0-mm-3D_glcm_JointEntropy | 0.992 |
| log-sigma-5-0-mm-3D_glcm_Imc1 | 0.941 |
| log-sigma-5-0-mm-3D_glcm_Imc2 | 0.954 |
| log-sigma-5-0-mm-3D_glcm_Id | 0.994 |
| log-sigma-5-0-mm-3D_glcm_Idn | 0.839 |
| log-sigma-5-0-mm-3D_glcm_SumEntropy | 0.987 |
| log-sigma-5-0-mm-3D_glcm_SumSquares | 0.994 |
| log-sigma-5-0-mm-3D_glrlm_GrayLevelNonUniformityNormalized | 0.985 |
| log-sigma-5-0-mm-3D_glrlm_HighGrayLevelRunEmphasis | 0.987 |
| log-sigma-5-0-mm-3D_glrlm_LongRunEmphasis | 0.953 |
| log-sigma-5-0-mm-3D_glrlm_LowGrayLevelRunEmphasis | 0.969 |
| log-sigma-5-0-mm-3D_glrlm_RunEntropy | 0.973 |
| log-sigma-5-0-mm-3D_glrlm_RunLengthNonUniformityNormalized | 0.991 |
| log-sigma-5-0-mm-3D_glrlm_RunVariance | 0.930 |
| log-sigma-5-0-mm-3D_glrlm_ShortRunEmphasis | 0.993 |
| log-sigma-5-0-mm-3D_glszm_GrayLevelNonUniformity | 0.854 |
| log-sigma-5-0-mm-3D_glszm_GrayLevelNonUniformityNormalized | 0.994 |
| log-sigma-5-0-mm-3D_glszm_GrayLevelVariance | 0.970 |
| log-sigma-5-0-mm-3D_glszm_LargeAreaLowGrayLevelEmphasis | 0.816 |
| log-sigma-5-0-mm-3D_glszm_LowGrayLevelZoneEmphasis | 0.973 |
| log-sigma-5-0-mm-3D_glszm_SmallAreaLowGrayLevelEmphasis | 0.849 |
| log-sigma-5-0-mm-3D_glszm_ZoneEntropy | 0.990 |
| log-sigma-5-0-mm-3D_glszm_ZonePercentage | 0.861 |
| log-sigma-5-0-mm-3D_gldm_DependenceNonUniformityNormalized | 0.991 |
| log-sigma-5-0-mm-3D_gldm_DependenceVariance | 0.920 |
| log-sigma-5-0-mm-3D_gldm_LargeDependenceEmphasis | 0.975 |
| log-sigma-5-0-mm-3D_gldm_LargeDependenceLowGrayLevelEmphasis | 0.957 |
| log-sigma-5-0-mm-3D_gldm_LowGrayLevelEmphasis | 0.948 |
| log-sigma-5-0-mm-3D_gldm_SmallDependenceHighGrayLevelEmphasis | 0.968 |
| log-sigma-5-0-mm-3D_gldm_SmallDependenceLowGrayLevelEmphasis | 0.958 |
| wavelet-LLH_firstorder_Kurtosis | 0.809 |
| wavelet-LLH_firstorder_Maximum | 0.880 |
| wavelet-LLH_firstorder_MeanAbsoluteDeviation | 0.847 |
| wavelet-LLH_firstorder_Minimum | 0.949 |
| wavelet-LLH_firstorder_Range | 0.950 |
| wavelet-LLH_firstorder_Variance | 0.809 |
| wavelet-LLH_glcm_Autocorrelation | 0.892 |
| wavelet-LLH_glcm_JointAverage | 0.943 |
| wavelet-LLH_glcm_Correlation | 0.929 |
| wavelet-LLH_glcm_DifferenceAverage | 0.907 |
| wavelet-LLH_glcm_JointEnergy | 0.991 |
| wavelet-LLH_glcm_JointEntropy | 0.988 |
| wavelet-LLH_glcm_Imc2 | 0.884 |
| wavelet-LLH_glcm_Idmn | 0.913 |
| wavelet-LLH_glcm_Id | 0.937 |
| wavelet-LLH_glcm_MaximumProbability | 0.965 |
| wavelet-LLH_glcm_SumEntropy | 0.971 |
| wavelet-LLH_glcm_SumSquares | 0.830 |
| wavelet-LLH_glrlm_GrayLevelVariance | 0.824 |
| wavelet-LLH_glrlm_HighGrayLevelRunEmphasis | 0.898 |
| wavelet-LLH_glrlm_LongRunLowGrayLevelEmphasis | 0.959 |
| wavelet-LLH_glrlm_LowGrayLevelRunEmphasis | 0.969 |
| wavelet-LLH_glrlm_RunEntropy | 0.966 |
| wavelet-LLH_glrlm_RunPercentage | 0.917 |
| wavelet-LLH_glrlm_RunVariance | 0.924 |
| wavelet-LLH_glrlm_ShortRunLowGrayLevelEmphasis | 0.971 |
| wavelet-LLH_glszm_GrayLevelNonUniformityNormalized | 0.891 |
| wavelet-LLH_glszm_GrayLevelVariance | 0.912 |
| wavelet-LLH_glszm_LargeAreaEmphasis | 0.983 |
| wavelet-LLH_glszm_LargeAreaHighGrayLevelEmphasis | 0.846 |
| wavelet-LLH_glszm_SizeZoneNonUniformityNormalized | 0.851 |
| wavelet-LLH_glszm_SmallAreaEmphasis | 0.836 |
| wavelet-LLH_glszm_SmallAreaHighGrayLevelEmphasis | 0.908 |
| wavelet-LLH_glszm_ZoneEntropy | 0.977 |
| wavelet-LLH_glszm_ZonePercentage | 0.845 |
| wavelet-LLH_gldm_DependenceEntropy | 0.972 |
| wavelet-LLH_gldm_DependenceVariance | 0.924 |
| wavelet-LLH_gldm_GrayLevelVariance | 0.809 |
| wavelet-LLH_gldm_HighGrayLevelEmphasis | 0.898 |
| wavelet-LLH_gldm_LargeDependenceLowGrayLevelEmphasis | 0.928 |
| wavelet-LLH_gldm_LowGrayLevelEmphasis | 0.966 |
| wavelet-LLH_gldm_SmallDependenceLowGrayLevelEmphasis | 0.885 |
| wavelet-LHL_firstorder_10Percentile | 0.948 |
| wavelet-LHL_firstorder_90Percentile | 0.969 |
| wavelet-LHL_firstorder_InterquartileRange | 0.924 |
| wavelet-LHL_firstorder_Kurtosis | 0.968 |
| wavelet-LHL_firstorder_Minimum | 0.966 |
| wavelet-LHL_firstorder_Range | 0.993 |
| wavelet-LHL_firstorder_RobustMeanAbsoluteDeviation | 0.932 |
| wavelet-LHL_firstorder_Skewness | 0.973 |
| wavelet-LHL_firstorder_Uniformity | 0.969 |
| wavelet-LHL_glcm_JointAverage | 0.971 |
| wavelet-LHL_glcm_ClusterProminence | 0.947 |
| wavelet-LHL_glcm_ClusterShade | 0.852 |
| wavelet-LHL_glcm_Contrast | 0.950 |
| wavelet-LHL_glcm_Correlation | 0.993 |
| wavelet-LHL_glcm_DifferenceVariance | 0.973 |
| wavelet-LHL_glcm_JointEnergy | 0.972 |
| wavelet-LHL_glcm_JointEntropy | 0.992 |
| wavelet-LHL_glcm_Imc1 | 0.947 |
| wavelet-LHL_glcm_Imc2 | 0.898 |
| wavelet-LHL_glcm_Idm | 0.961 |
| wavelet-LHL_glcm_Idn | 0.892 |
| wavelet-LHL_glcm_InverseVariance | 0.964 |
| wavelet-LHL_glcm_MaximumProbability | 0.913 |
| wavelet-LHL_glcm_SumSquares | 0.955 |
| wavelet-LHL_glrlm_GrayLevelNonUniformityNormalized | 0.974 |
| wavelet-LHL_glrlm_LongRunEmphasis | 0.929 |
| wavelet-LHL_glrlm_LongRunHighGrayLevelEmphasis | 0.951 |
| wavelet-LHL_glrlm_LongRunLowGrayLevelEmphasis | 0.991 |
| wavelet-LHL_glrlm_RunEntropy | 0.988 |
| wavelet-LHL_glrlm_RunLengthNonUniformityNormalized | 0.940 |
| wavelet-LHL_glrlm_ShortRunEmphasis | 0.937 |
| wavelet-LHL_glrlm_ShortRunHighGrayLevelEmphasis | 0.954 |
| wavelet-LHL_glrlm_ShortRunLowGrayLevelEmphasis | 0.988 |
| wavelet-LHL_glszm_GrayLevelVariance | 0.981 |
| wavelet-LHL_glszm_HighGrayLevelZoneEmphasis | 0.954 |
| wavelet-LHL_glszm_LargeAreaLowGrayLevelEmphasis | 0.969 |
| wavelet-LHL_glszm_LowGrayLevelZoneEmphasis | 0.990 |
| wavelet-LHL_glszm_SizeZoneNonUniformity | 0.814 |
| wavelet-LHL_glszm_SmallAreaEmphasis | 0.952 |
| wavelet-LHL_glszm_SmallAreaHighGrayLevelEmphasis | 0.960 |
| wavelet-LHL_glszm_ZonePercentage | 0.962 |
| wavelet-LHL_glszm_ZoneVariance | 0.933 |
| wavelet-LHL_gldm_DependenceEntropy | 0.984 |
| wavelet-LHL_gldm_DependenceVariance | 0.809 |
| wavelet-LHL_gldm_GrayLevelVariance | 0.959 |
| wavelet-LHL_gldm_LargeDependenceHighGrayLevelEmphasis | 0.905 |
| wavelet-LHL_gldm_LargeDependenceLowGrayLevelEmphasis | 0.989 |
| wavelet-LHL_gldm_LowGrayLevelEmphasis | 0.987 |
| wavelet-LHL_gldm_SmallDependenceHighGrayLevelEmphasis | 0.954 |
| wavelet-LHL_gldm_SmallDependenceLowGrayLevelEmphasis | 0.985 |
| wavelet-LHH_firstorder_Entropy | 0.992 |
| wavelet-LHH_firstorder_InterquartileRange | 0.956 |
| wavelet-LHH_firstorder_Kurtosis | 0.871 |
| wavelet-LHH_firstorder_MeanAbsoluteDeviation | 0.977 |
| wavelet-LHH_firstorder_Mean | 0.854 |
| wavelet-LHH_firstorder_RobustMeanAbsoluteDeviation | 0.971 |
| wavelet-LHH_firstorder_RootMeanSquared | 0.933 |
| wavelet-LHH_firstorder_Skewness | 0.945 |
| wavelet-LHH_firstorder_Variance | 0.968 |
| wavelet-LHH_glcm_Autocorrelation | 0.874 |
| wavelet-LHH_glcm_ClusterShade | 0.986 |
| wavelet-LHH_glcm_ClusterTendency | 0.966 |
| wavelet-LHH_glcm_Contrast | 0.964 |
| wavelet-LHH_glcm_DifferenceAverage | 0.979 |
| wavelet-LHH_glcm_DifferenceEntropy | 0.994 |
| wavelet-LHH_glcm_JointEntropy | 0.995 |
| wavelet-LHH_glcm_Imc2 | 0.906 |
| wavelet-LHH_glcm_Idm | 0.988 |
| wavelet-LHH_glcm_Id | 0.987 |
| wavelet-LHH_glcm_Idn | 0.825 |
| wavelet-LHH_glcm_SumEntropy | 0.994 |
| wavelet-LHH_glcm_SumSquares | 0.965 |
| wavelet-LHH_glrlm_GrayLevelNonUniformityNormalized | 0.989 |
| wavelet-LHH_glrlm_HighGrayLevelRunEmphasis | 0.885 |
| wavelet-LHH_glrlm_LongRunEmphasis | 0.942 |
| wavelet-LHH_glrlm_LowGrayLevelRunEmphasis | 0.893 |
| wavelet-LHH_glrlm_RunEntropy | 0.995 |
| wavelet-LHH_glrlm_RunLengthNonUniformityNormalized | 0.968 |
| wavelet-LHH_glrlm_RunVariance | 0.936 |
| wavelet-LHH_glrlm_ShortRunEmphasis | 0.963 |
| wavelet-LHH_glszm_GrayLevelNonUniformityNormalized | 0.999 |
| wavelet-LHH_glszm_GrayLevelVariance | 0.994 |
| wavelet-LHH_glszm_HighGrayLevelZoneEmphasis | 0.886 |
| wavelet-LHH_glszm_LargeAreaHighGrayLevelEmphasis | 0.886 |
| wavelet-LHH_glszm_LargeAreaLowGrayLevelEmphasis | 0.931 |
| wavelet-LHH_glszm_SmallAreaEmphasis | 0.879 |
| wavelet-LHH_glszm_SmallAreaHighGrayLevelEmphasis | 0.900 |
| wavelet-LHH_glszm_SmallAreaLowGrayLevelEmphasis | 0.904 |
| wavelet-LHH_glszm_ZonePercentage | 0.954 |
| wavelet-LHH_glszm_ZoneVariance | 0.937 |
| wavelet-LHH_gldm_DependenceVariance | 0.896 |
| wavelet-LHH_gldm_GrayLevelVariance | 0.968 |
| wavelet-LHH_gldm_HighGrayLevelEmphasis | 0.884 |
| wavelet-LHH_gldm_LargeDependenceLowGrayLevelEmphasis | 0.932 |
| wavelet-LHH_gldm_LowGrayLevelEmphasis | 0.897 |
| wavelet-LHH_gldm_SmallDependenceLowGrayLevelEmphasis | 0.866 |
| wavelet-HLL_firstorder_10Percentile | 0.957 |
| wavelet-HLL_firstorder_90Percentile | 0.976 |
| wavelet-HLL_firstorder_InterquartileRange | 0.925 |
| wavelet-HLL_firstorder_Kurtosis | 0.959 |
| wavelet-HLL_firstorder_Mean | 0.862 |
| wavelet-HLL_firstorder_Median | 0.839 |
| wavelet-HLL_firstorder_Minimum | 0.885 |
| wavelet-HLL_firstorder_RobustMeanAbsoluteDeviation | 0.943 |
| wavelet-HLL_firstorder_RootMeanSquared | 0.903 |
| wavelet-HLL_firstorder_Variance | 0.970 |
| wavelet-HLL_glcm_JointAverage | 0.881 |
| wavelet-HLL_glcm_ClusterProminence | 0.923 |
| wavelet-HLL_glcm_Contrast | 0.963 |
| wavelet-HLL_glcm_Correlation | 0.980 |
| wavelet-HLL_glcm_DifferenceVariance | 0.976 |
| wavelet-HLL_glcm_JointEnergy | 0.980 |
| wavelet-HLL_glcm_JointEntropy | 0.987 |
| wavelet-HLL_glcm_Idm | 0.970 |
| wavelet-HLL_glcm_Idmn | 0.898 |
| wavelet-HLL_glcm_InverseVariance | 0.970 |
| wavelet-HLL_glcm_MaximumProbability | 0.964 |
| wavelet-HLL_glcm_SumEntropy | 0.981 |
| wavelet-HLL_glrlm_GrayLevelNonUniformityNormalized | 0.976 |
| wavelet-HLL_glrlm_GrayLevelVariance | 0.972 |
| wavelet-HLL_glrlm_LowGrayLevelRunEmphasis | 0.995 |
| wavelet-HLL_glrlm_RunEntropy | 0.985 |
| wavelet-HLL_glrlm_RunLengthNonUniformityNormalized | 0.964 |
| wavelet-HLL_glrlm_RunVariance | 0.957 |
| wavelet-HLL_glrlm_ShortRunEmphasis | 0.967 |
| wavelet-HLL_glszm_GrayLevelVariance | 0.979 |
| wavelet-HLL_glszm_LargeAreaEmphasis | 0.989 |
| wavelet-HLL_glszm_LargeAreaHighGrayLevelEmphasis | 0.856 |
| wavelet-HLL_glszm_LowGrayLevelZoneEmphasis | 0.993 |
| wavelet-HLL_glszm_SizeZoneNonUniformityNormalized | 0.971 |
| wavelet-HLL_glszm_ZoneEntropy | 0.984 |
| wavelet-HLL_glszm_ZonePercentage | 0.954 |
| wavelet-HLL_glszm_ZoneVariance | 0.989 |
| wavelet-HLL_gldm_DependenceNonUniformity | 0.943 |
| wavelet-HLL_gldm_DependenceNonUniformityNormalized | 0.923 |
| wavelet-HLL_gldm_LargeDependenceEmphasis | 0.956 |
| wavelet-HLL_gldm_LargeDependenceLowGrayLevelEmphasis | 0.992 |
| wavelet-HLL_gldm_LowGrayLevelEmphasis | 0.995 |
| wavelet-HLL_gldm_SmallDependenceLowGrayLevelEmphasis | 0.940 |
| wavelet-HLH_firstorder_10Percentile | 0.954 |
| wavelet-HLH_firstorder_InterquartileRange | 0.945 |
| wavelet-HLH_firstorder_Kurtosis | 0.808 |
| wavelet-HLH_firstorder_Maximum | 0.943 |
| wavelet-HLH_firstorder_Minimum | 0.986 |
| wavelet-HLH_firstorder_Range | 0.978 |
| wavelet-HLH_firstorder_Skewness | 0.921 |
| wavelet-HLH_firstorder_Uniformity | 0.985 |
| wavelet-HLH_firstorder_Variance | 0.968 |
| wavelet-HLH_glcm_JointAverage | 0.989 |
| wavelet-HLH_glcm_ClusterProminence | 0.932 |
| wavelet-HLH_glcm_Correlation | 0.962 |
| wavelet-HLH_glcm_DifferenceAverage | 0.975 |
| wavelet-HLH_glcm_DifferenceEntropy | 0.992 |
| wavelet-HLH_glcm_JointEnergy | 0.991 |
| wavelet-HLH_glcm_JointEntropy | 0.994 |
| wavelet-HLH_glcm_Imc1 | 0.814 |
| wavelet-HLH_glcm_Idm | 0.985 |
| wavelet-HLH_glcm_Id | 0.984 |
| wavelet-HLH_glcm_InverseVariance | 0.982 |
| wavelet-HLH_glcm_SumEntropy | 0.993 |
| wavelet-HLH_glcm_SumSquares | 0.966 |
| wavelet-HLH_glrlm_HighGrayLevelRunEmphasis | 0.985 |
| wavelet-HLH_glrlm_LongRunEmphasis | 0.972 |
| wavelet-HLH_glrlm_LongRunHighGrayLevelEmphasis | 0.981 |
| wavelet-HLH_glrlm_LowGrayLevelRunEmphasis | 0.993 |
| wavelet-HLH_glrlm_RunEntropy | 0.995 |
| wavelet-HLH_glrlm_RunVariance | 0.972 |
| wavelet-HLH_glrlm_ShortRunEmphasis | 0.971 |
| wavelet-HLH_glrlm_ShortRunHighGrayLevelEmphasis | 0.985 |
| wavelet-HLH_glszm_GrayLevelNonUniformityNormalized | 0.989 |
| wavelet-HLH_glszm_GrayLevelVariance | 0.981 |
| wavelet-HLH_glszm_LargeAreaLowGrayLevelEmphasis | 0.964 |
| wavelet-HLH_glszm_LowGrayLevelZoneEmphasis | 0.972 |
| wavelet-HLH_glszm_SizeZoneNonUniformity | 0.986 |
| wavelet-HLH_glszm_SmallAreaEmphasis | 0.815 |
| wavelet-HLH_glszm_SmallAreaHighGrayLevelEmphasis | 0.983 |
| wavelet-HLH_glszm_ZonePercentage | 0.916 |
| wavelet-HLH_glszm_ZoneVariance | 0.932 |
| wavelet-HLH_gldm_DependenceEntropy | 0.987 |
| wavelet-HLH_gldm_DependenceVariance | 0.973 |
| wavelet-HLH_gldm_GrayLevelVariance | 0.968 |
| wavelet-HLH_gldm_LargeDependenceHighGrayLevelEmphasis | 0.948 |
| wavelet-HLH_gldm_LargeDependenceLowGrayLevelEmphasis | 0.988 |
| wavelet-HLH_gldm_LowGrayLevelEmphasis | 0.994 |
| wavelet-HLH_gldm_SmallDependenceHighGrayLevelEmphasis | 0.968 |
| wavelet-HLH_gldm_SmallDependenceLowGrayLevelEmphasis | 0.870 |
| wavelet-HHL_firstorder_Entropy | 0.997 |
| wavelet-HHL_firstorder_InterquartileRange | 0.992 |
| wavelet-HHL_firstorder_Kurtosis | 0.987 |
| wavelet-HHL_firstorder_MeanAbsoluteDeviation | 0.995 |
| wavelet-HHL_firstorder_Minimum | 0.985 |
| wavelet-HHL_firstorder_RootMeanSquared | 0.982 |
| wavelet-HHL_firstorder_Skewness | 0.943 |
| wavelet-HHL_firstorder_Uniformity | 0.994 |
| wavelet-HHL_glcm_Autocorrelation | 0.966 |
| wavelet-HHL_glcm_JointAverage | 0.983 |
| wavelet-HHL_glcm_Contrast | 0.993 |
| wavelet-HHL_glcm_Correlation | 0.944 |
| wavelet-HHL_glcm_DifferenceAverage | 0.993 |
| wavelet-HHL_glcm_DifferenceVariance | 0.995 |
| wavelet-HHL_glcm_JointEnergy | 0.988 |
| wavelet-HHL_glcm_Idm | 0.994 |
| wavelet-HHL_glcm_Idmn | 0.946 |
| wavelet-HHL_glcm_Id | 0.994 |
| wavelet-HHL_glcm_InverseVariance | 0.996 |
| wavelet-HHL_glcm_MaximumProbability | 0.984 |
| wavelet-HHL_glrlm_GrayLevelNonUniformityNormalized | 0.996 |
| wavelet-HHL_glrlm_GrayLevelVariance | 0.996 |
| wavelet-HHL_glrlm_HighGrayLevelRunEmphasis | 0.969 |

**TABLE 3:** Radiomic Features with ICC≥0.8 in Contrast Enhanced CT Image

| **Radiomic Feature** | **ICC value** |
| --- | --- |
| original_shape_LeastAxisLength | 0.971 |
| original_shape_MajorAxisLength | 0.934 |
| original_shape_Maximum2DDiameterColumn | 0.935 |
| original_shape_Maximum2DDiameterSlice | 0.974 |
| original_shape_Maximum3DDiameter | 0.956 |
| original_shape_Sphericity | 0.857 |
| original_shape_SurfaceArea | 0.948 |
| original_shape_SurfaceVolumeRatio | 0.914 |
| original_firstorder_10Percentile | 0.920 |
| original_firstorder_Energy | 0.966 |
| original_firstorder_Entropy | 0.972 |
| original_firstorder_Maximum | 0.949 |
| original_firstorder_MeanAbsoluteDeviation | 0.980 |
| original_firstorder_Mean | 0.960 |
| original_firstorder_Minimum | 0.906 |
| original_firstorder_Range | 0.954 |
| original_firstorder_Skewness | 0.936 |
| original_firstorder_TotalEnergy | 0.966 |
| original_firstorder_Uniformity | 0.978 |
| original_glcm_Autocorrelation | 0.972 |
| original_glcm_JointAverage | 0.961 |
| original_glcm_ClusterShade | 0.890 |
| original_glcm_ClusterTendency | 0.969 |
| original_glcm_Contrast | 0.968 |
| original_glcm_Correlation | 0.955 |
| original_glcm_DifferenceEntropy | 0.979 |
| original_glcm_DifferenceVariance | 0.983 |
| original_glcm_Imc1 | 0.884 |
| original_glcm_Imc2 | 0.951 |
| original_glcm_Idm | 0.959 |
| original_glcm_Id | 0.956 |
| original_glcm_Idn | 0.900 |
| original_glcm_SumEntropy | 0.983 |
| original_glcm_SumSquares | 0.973 |
| original_glrlm_GrayLevelNonUniformityNormalized | 0.979 |
| original_glrlm_GrayLevelVariance | 0.975 |
| original_glrlm_HighGrayLevelRunEmphasis | 0.972 |
| original_glrlm_LongRunEmphasis | 0.965 |
| original_glrlm_LowGrayLevelRunEmphasis | 0.852 |
| original_glrlm_RunEntropy | 0.982 |
| original_glrlm_RunLengthNonUniformityNormalized | 0.937 |
| original_glrlm_RunVariance | 0.975 |
| original_glrlm_ShortRunEmphasis | 0.943 |
| original_glszm_GrayLevelNonUniformityNormalized | 0.991 |
| original_glszm_GrayLevelVariance | 0.975 |
| original_glszm_HighGrayLevelZoneEmphasis | 0.970 |
| original_glszm_LargeAreaHighGrayLevelEmphasis | 0.995 |
| original_glszm_LargeAreaLowGrayLevelEmphasis | 0.817 |
| original_glszm_LowGrayLevelZoneEmphasis | 0.952 |
| original_glszm_SizeZoneNonUniformity | 0.867 |
| original_glszm_SizeZoneNonUniformityNormalized | 0.881 |
| original_glszm_SmallAreaEmphasis | 0.878 |
| original_glszm_SmallAreaHighGrayLevelEmphasis | 0.966 |
| original_glszm_ZoneEntropy | 0.952 |
| original_glszm_ZonePercentage | 0.878 |
| original_gldm_DependenceNonUniformity | 0.813 |
| original_gldm_DependenceNonUniformityNormalized | 0.922 |
| original_gldm_DependenceVariance | 0.994 |
| original_gldm_HighGrayLevelEmphasis | 0.972 |
| original_gldm_LargeDependenceEmphasis | 0.980 |
| original_gldm_SmallDependenceEmphasis | 0.868 |
| original_gldm_SmallDependenceHighGrayLevelEmphasis | 0.927 |
| original_gldm_SmallDependenceLowGrayLevelEmphasis | 0.975 |
| log-sigma-1-0-mm-3D_firstorder_90Percentile | 0.879 |
| log-sigma-1-0-mm-3D_firstorder_Entropy | 0.984 |
| log-sigma-1-0-mm-3D_firstorder_Maximum | 0.981 |
| log-sigma-1-0-mm-3D_firstorder_MeanAbsoluteDeviation | 0.970 |
| log-sigma-1-0-mm-3D_firstorder_Minimum | 0.989 |
| log-sigma-1-0-mm-3D_firstorder_RobustMeanAbsoluteDeviation | 0.961 |
| log-sigma-1-0-mm-3D_firstorder_Skewness | 0.873 |
| log-sigma-1-0-mm-3D_glcm_Autocorrelation | 0.964 |
| log-sigma-1-0-mm-3D_glcm_JointAverage | 0.980 |
| log-sigma-1-0-mm-3D_glcm_ClusterProminence | 0.945 |
| log-sigma-1-0-mm-3D_glcm_Contrast | 0.960 |
| log-sigma-1-0-mm-3D_glcm_Correlation | 0.897 |
| log-sigma-1-0-mm-3D_glcm_DifferenceVariance | 0.974 |
| log-sigma-1-0-mm-3D_glcm_JointEnergy | 0.994 |
| log-sigma-1-0-mm-3D_glcm_JointEntropy | 0.989 |
| log-sigma-1-0-mm-3D_glcm_Imc2 | 0.951 |
| log-sigma-1-0-mm-3D_glcm_Idm | 0.967 |
| log-sigma-1-0-mm-3D_glcm_Idn | 0.967 |
| log-sigma-1-0-mm-3D_glcm_InverseVariance | 0.964 |
| log-sigma-1-0-mm-3D_glcm_MaximumProbability | 0.987 |
| log-sigma-1-0-mm-3D_glcm_SumSquares | 0.965 |
| log-sigma-1-0-mm-3D_glrlm_GrayLevelNonUniformityNormalized | 0.985 |
| log-sigma-1-0-mm-3D_glrlm_LongRunEmphasis | 0.962 |
| log-sigma-1-0-mm-3D_glrlm_LongRunHighGrayLevelEmphasis | 0.952 |
| log-sigma-1-0-mm-3D_glrlm_LongRunLowGrayLevelEmphasis | 0.996 |
| log-sigma-1-0-mm-3D_glrlm_RunEntropy | 0.988 |
| log-sigma-1-0-mm-3D_glrlm_RunLengthNonUniformityNormalized | 0.943 |
| log-sigma-1-0-mm-3D_glrlm_ShortRunEmphasis | 0.951 |
| log-sigma-1-0-mm-3D_glrlm_ShortRunHighGrayLevelEmphasis | 0.973 |
| log-sigma-1-0-mm-3D_glrlm_ShortRunLowGrayLevelEmphasis | 0.989 |
| log-sigma-1-0-mm-3D_glszm_GrayLevelNonUniformityNormalized | 0.988 |
| log-sigma-1-0-mm-3D_glszm_GrayLevelVariance | 0.968 |
| log-sigma-1-0-mm-3D_glszm_LargeAreaHighGrayLevelEmphasis | 0.975 |
| log-sigma-1-0-mm-3D_glszm_LargeAreaLowGrayLevelEmphasis | 0.997 |
| log-sigma-1-0-mm-3D_glszm_LowGrayLevelZoneEmphasis | 0.992 |
| log-sigma-1-0-mm-3D_glszm_SizeZoneNonUniformityNormalized | 0.977 |
| log-sigma-1-0-mm-3D_glszm_SmallAreaEmphasis | 0.981 |
| log-sigma-1-0-mm-3D_glszm_ZoneEntropy | 0.986 |
| log-sigma-1-0-mm-3D_glszm_ZonePercentage | 0.909 |
| log-sigma-1-0-mm-3D_glszm_ZoneVariance | 0.991 |
| log-sigma-1-0-mm-3D_gldm_DependenceNonUniformity | 0.800 |
| log-sigma-1-0-mm-3D_gldm_DependenceNonUniformityNormalized | 0.938 |
| log-sigma-1-0-mm-3D_gldm_HighGrayLevelEmphasis | 0.968 |
| log-sigma-1-0-mm-3D_gldm_LargeDependenceEmphasis | 0.966 |
| log-sigma-1-0-mm-3D_gldm_LargeDependenceHighGrayLevelEmphasis | 0.936 |
| log-sigma-1-0-mm-3D_gldm_LowGrayLevelEmphasis | 0.992 |
| log-sigma-1-0-mm-3D_gldm_SmallDependenceEmphasis | 0.914 |
| log-sigma-2-0-mm-3D_firstorder_10Percentile | 0.950 |
| log-sigma-2-0-mm-3D_firstorder_90Percentile | 0.880 |
| log-sigma-2-0-mm-3D_firstorder_Entropy | 0.989 |
| log-sigma-2-0-mm-3D_firstorder_Maximum | 0.984 |
| log-sigma-2-0-mm-3D_firstorder_MeanAbsoluteDeviation | 0.983 |
| log-sigma-2-0-mm-3D_firstorder_Minimum | 0.991 |
| log-sigma-2-0-mm-3D_firstorder_Range | 0.989 |
| log-sigma-2-0-mm-3D_firstorder_RobustMeanAbsoluteDeviation | 0.962 |
| log-sigma-2-0-mm-3D_firstorder_Skewness | 0.911 |
| log-sigma-2-0-mm-3D_firstorder_Uniformity | 0.988 |
| log-sigma-2-0-mm-3D_glcm_JointAverage | 0.925 |
| log-sigma-2-0-mm-3D_glcm_ClusterProminence | 0.984 |
| log-sigma-2-0-mm-3D_glcm_ClusterShade | 0.896 |
| log-sigma-2-0-mm-3D_glcm_Contrast | 0.986 |
| log-sigma-2-0-mm-3D_glcm_Correlation | 0.946 |
| log-sigma-2-0-mm-3D_glcm_DifferenceVariance | 0.992 |
| log-sigma-2-0-mm-3D_glcm_JointEnergy | 0.992 |
| log-sigma-2-0-mm-3D_glcm_JointEntropy | 0.990 |
| log-sigma-2-0-mm-3D_glcm_Imc2 | 0.939 |
| log-sigma-2-0-mm-3D_glcm_Idm | 0.966 |
| log-sigma-2-0-mm-3D_glcm_Idn | 0.917 |
| log-sigma-2-0-mm-3D_glcm_InverseVariance | 0.980 |
| log-sigma-2-0-mm-3D_glcm_MaximumProbability | 0.970 |
| log-sigma-2-0-mm-3D_glcm_SumSquares | 0.991 |
| log-sigma-2-0-mm-3D_glrlm_GrayLevelNonUniformityNormalized | 0.989 |
| log-sigma-2-0-mm-3D_glrlm_LongRunEmphasis | 0.943 |
| log-sigma-2-0-mm-3D_glrlm_LongRunLowGrayLevelEmphasis | 0.993 |
| log-sigma-2-0-mm-3D_glrlm_LowGrayLevelRunEmphasis | 0.981 |
| log-sigma-2-0-mm-3D_glrlm_RunLengthNonUniformityNormalized | 0.936 |
| log-sigma-2-0-mm-3D_glrlm_RunPercentage | 0.935 |
| log-sigma-2-0-mm-3D_glrlm_ShortRunHighGrayLevelEmphasis | 0.929 |
| log-sigma-2-0-mm-3D_glrlm_ShortRunLowGrayLevelEmphasis | 0.973 |
| log-sigma-2-0-mm-3D_glszm_GrayLevelNonUniformity | 0.938 |
| log-sigma-2-0-mm-3D_glszm_GrayLevelVariance | 0.964 |
| log-sigma-2-0-mm-3D_glszm_HighGrayLevelZoneEmphasis | 0.963 |
| log-sigma-2-0-mm-3D_glszm_LargeAreaLowGrayLevelEmphasis | 0.972 |
| log-sigma-2-0-mm-3D_glszm_LowGrayLevelZoneEmphasis | 0.992 |
| log-sigma-2-0-mm-3D_glszm_SizeZoneNonUniformity | 0.939 |
| log-sigma-2-0-mm-3D_glszm_SmallAreaEmphasis | 0.973 |
| log-sigma-2-0-mm-3D_glszm_SmallAreaHighGrayLevelEmphasis | 0.982 |
| log-sigma-2-0-mm-3D_glszm_ZonePercentage | 0.942 |
| log-sigma-2-0-mm-3D_glszm_ZoneVariance | 0.979 |
| log-sigma-2-0-mm-3D_gldm_DependenceEntropy | 0.964 |
| log-sigma-2-0-mm-3D_gldm_DependenceVariance | 0.821 |
| log-sigma-2-0-mm-3D_gldm_GrayLevelVariance | 0.991 |
| log-sigma-2-0-mm-3D_gldm_LargeDependenceLowGrayLevelEmphasis | 0.998 |
| log-sigma-2-0-mm-3D_gldm_LowGrayLevelEmphasis | 0.984 |
| log-sigma-2-0-mm-3D_gldm_SmallDependenceEmphasis | 0.951 |
| log-sigma-2-0-mm-3D_gldm_SmallDependenceLowGrayLevelEmphasis | 0.935 |
| log-sigma-3-0-mm-3D_firstorder_10Percentile | 0.987 |
| log-sigma-3-0-mm-3D_firstorder_InterquartileRange | 0.967 |
| log-sigma-3-0-mm-3D_firstorder_Maximum | 0.977 |
| log-sigma-3-0-mm-3D_firstorder_MeanAbsoluteDeviation | 0.994 |
| log-sigma-3-0-mm-3D_firstorder_Median | 0.916 |
| log-sigma-3-0-mm-3D_firstorder_Minimum | 0.997 |
| log-sigma-3-0-mm-3D_firstorder_RootMeanSquared | 0.954 |
| log-sigma-3-0-mm-3D_firstorder_Skewness | 0.801 |
| log-sigma-3-0-mm-3D_firstorder_Uniformity | 0.992 |
| log-sigma-3-0-mm-3D_glcm_Autocorrelation | 0.961 |
| log-sigma-3-0-mm-3D_glcm_JointAverage | 0.969 |
| log-sigma-3-0-mm-3D_glcm_ClusterTendency | 0.998 |
| log-sigma-3-0-mm-3D_glcm_Contrast | 0.997 |
| log-sigma-3-0-mm-3D_glcm_Correlation | 0.977 |
| log-sigma-3-0-mm-3D_glcm_DifferenceEntropy | 0.998 |
| log-sigma-3-0-mm-3D_glcm_DifferenceVariance | 0.998 |
| log-sigma-3-0-mm-3D_glcm_Imc1 | 0.942 |
| log-sigma-3-0-mm-3D_glcm_Imc2 | 0.962 |
| log-sigma-3-0-mm-3D_glcm_Idm | 0.987 |
| log-sigma-3-0-mm-3D_glcm_Id | 0.987 |
| log-sigma-3-0-mm-3D_glcm_Idn | 0.924 |
| log-sigma-3-0-mm-3D_glcm_SumEntropy | 0.995 |
| log-sigma-3-0-mm-3D_glcm_SumSquares | 0.998 |
| log-sigma-3-0-mm-3D_glrlm_GrayLevelNonUniformityNormalized | 0.992 |
| log-sigma-3-0-mm-3D_glrlm_HighGrayLevelRunEmphasis | 0.975 |
| log-sigma-3-0-mm-3D_glrlm_LongRunEmphasis | 0.939 |
| log-sigma-3-0-mm-3D_glrlm_LowGrayLevelRunEmphasis | 0.873 |
| log-sigma-3-0-mm-3D_glrlm_RunEntropy | 0.981 |
| log-sigma-3-0-mm-3D_glrlm_RunLengthNonUniformityNormalized | 0.975 |
| log-sigma-3-0-mm-3D_glrlm_RunVariance | 0.905 |
| log-sigma-3-0-mm-3D_glrlm_ShortRunEmphasis | 0.978 |
| log-sigma-3-0-mm-3D_glszm_GrayLevelNonUniformity | 0.993 |
| log-sigma-3-0-mm-3D_glszm_GrayLevelNonUniformityNormalized | 0.965 |
| log-sigma-3-0-mm-3D_glszm_GrayLevelVariance | 0.980 |
| log-sigma-3-0-mm-3D_glszm_LargeAreaLowGrayLevelEmphasis | 0.937 |
| log-sigma-3-0-mm-3D_glszm_LowGrayLevelZoneEmphasis | 0.970 |
| log-sigma-3-0-mm-3D_glszm_ZoneEntropy | 0.981 |
| log-sigma-3-0-mm-3D_glszm_ZonePercentage | 0.981 |
| log-sigma-3-0-mm-3D_gldm_DependenceEntropy | 0.971 |
| log-sigma-3-0-mm-3D_gldm_DependenceNonUniformityNormalized | 0.960 |
| log-sigma-3-0-mm-3D_gldm_DependenceVariance | 0.858 |
| log-sigma-3-0-mm-3D_gldm_LargeDependenceEmphasis | 0.945 |
| log-sigma-3-0-mm-3D_gldm_LowGrayLevelEmphasis | 0.830 |
| log-sigma-3-0-mm-3D_gldm_SmallDependenceEmphasis | 0.983 |
| log-sigma-3-0-mm-3D_gldm_SmallDependenceLowGrayLevelEmphasis | 0.815 |
| log-sigma-4-0-mm-3D_firstorder_10Percentile | 0.995 |
| log-sigma-4-0-mm-3D_firstorder_InterquartileRange | 0.992 |
| log-sigma-4-0-mm-3D_firstorder_Maximum | 0.970 |
| log-sigma-4-0-mm-3D_firstorder_MeanAbsoluteDeviation | 0.998 |
| log-sigma-4-0-mm-3D_firstorder_Median | 0.966 |
| log-sigma-4-0-mm-3D_firstorder_Minimum | 0.998 |
| log-sigma-4-0-mm-3D_firstorder_RootMeanSquared | 0.982 |
| log-sigma-4-0-mm-3D_firstorder_Skewness | 0.828 |
| log-sigma-4-0-mm-3D_firstorder_Uniformity | 0.987 |
| log-sigma-4-0-mm-3D_glcm_Autocorrelation | 0.978 |
| log-sigma-4-0-mm-3D_glcm_JointAverage | 0.982 |
| log-sigma-4-0-mm-3D_glcm_ClusterTendency | 0.998 |
| log-sigma-4-0-mm-3D_glcm_Contrast | 0.998 |
| log-sigma-4-0-mm-3D_glcm_Correlation | 0.970 |
| log-sigma-4-0-mm-3D_glcm_DifferenceEntropy | 0.998 |
| log-sigma-4-0-mm-3D_glcm_DifferenceVariance | 0.998 |
| log-sigma-4-0-mm-3D_glcm_Imc1 | 0.925 |
| log-sigma-4-0-mm-3D_glcm_Imc2 | 0.963 |
| log-sigma-4-0-mm-3D_glcm_Idm | 0.993 |
| log-sigma-4-0-mm-3D_glcm_Id | 0.993 |
| log-sigma-4-0-mm-3D_glcm_Idn | 0.959 |
| log-sigma-4-0-mm-3D_glcm_SumEntropy | 0.996 |
| log-sigma-4-0-mm-3D_glcm_SumSquares | 0.998 |
| log-sigma-4-0-mm-3D_glrlm_GrayLevelNonUniformityNormalized | 0.990 |
| log-sigma-4-0-mm-3D_glrlm_HighGrayLevelRunEmphasis | 0.986 |
| log-sigma-4-0-mm-3D_glrlm_LongRunEmphasis | 0.973 |
| log-sigma-4-0-mm-3D_glrlm_LowGrayLevelRunEmphasis | 0.993 |
| log-sigma-4-0-mm-3D_glrlm_RunEntropy | 0.984 |
| log-sigma-4-0-mm-3D_glrlm_RunLengthNonUniformity | 0.834 |
| log-sigma-4-0-mm-3D_glrlm_RunPercentage | 0.981 |
| log-sigma-4-0-mm-3D_glrlm_RunVariance | 0.923 |
| log-sigma-4-0-mm-3D_glrlm_ShortRunLowGrayLevelEmphasis | 0.988 |
| log-sigma-4-0-mm-3D_glszm_GrayLevelNonUniformity | 0.968 |
| log-sigma-4-0-mm-3D_glszm_GrayLevelNonUniformityNormalized | 0.953 |
| log-sigma-4-0-mm-3D_glszm_HighGrayLevelZoneEmphasis | 0.995 |
| log-sigma-4-0-mm-3D_glszm_LargeAreaLowGrayLevelEmphasis | 0.924 |
| log-sigma-4-0-mm-3D_glszm_SizeZoneNonUniformityNormalized | 0.865 |
| log-sigma-4-0-mm-3D_glszm_SmallAreaEmphasis | 0.861 |
| log-sigma-4-0-mm-3D_glszm_SmallAreaHighGrayLevelEmphasis | 0.991 |
| log-sigma-4-0-mm-3D_glszm_ZoneEntropy | 0.986 |
| log-sigma-4-0-mm-3D_glszm_ZonePercentage | 0.967 |
| log-sigma-4-0-mm-3D_gldm_DependenceNonUniformityNormalized | 0.977 |
| log-sigma-4-0-mm-3D_gldm_DependenceVariance | 0.925 |
| log-sigma-4-0-mm-3D_gldm_GrayLevelVariance | 0.998 |
| log-sigma-4-0-mm-3D_gldm_LargeDependenceEmphasis | 0.980 |
| log-sigma-4-0-mm-3D_gldm_LargeDependenceLowGrayLevelEmphasis | 0.991 |
| log-sigma-4-0-mm-3D_gldm_SmallDependenceHighGrayLevelEmphasis | 0.995 |
| log-sigma-4-0-mm-3D_gldm_SmallDependenceLowGrayLevelEmphasis | 0.946 |
| log-sigma-5-0-mm-3D_firstorder_10Percentile | 0.997 |
| log-sigma-5-0-mm-3D_firstorder_Entropy | 0.997 |
| log-sigma-5-0-mm-3D_firstorder_InterquartileRange | 0.999 |
| log-sigma-5-0-mm-3D_firstorder_MeanAbsoluteDeviation | 0.999 |
| log-sigma-5-0-mm-3D_firstorder_Mean | 0.992 |
| log-sigma-5-0-mm-3D_firstorder_Median | 0.989 |
| log-sigma-5-0-mm-3D_firstorder_Range | 0.996 |
| log-sigma-5-0-mm-3D_firstorder_RobustMeanAbsoluteDeviation | 0.999 |
| log-sigma-5-0-mm-3D_firstorder_Uniformity | 0.991 |
| log-sigma-5-0-mm-3D_firstorder_Variance | 0.999 |
| log-sigma-5-0-mm-3D_glcm_Autocorrelation | 0.991 |
| log-sigma-5-0-mm-3D_glcm_ClusterProminence | 0.997 |
| log-sigma-5-0-mm-3D_glcm_ClusterShade | 0.931 |
| log-sigma-5-0-mm-3D_glcm_Correlation | 0.982 |
| log-sigma-5-0-mm-3D_glcm_DifferenceAverage | 0.998 |
| log-sigma-5-0-mm-3D_glcm_DifferenceEntropy | 0.998 |
| log-sigma-5-0-mm-3D_glcm_JointEnergy | 0.990 |
| log-sigma-5-0-mm-3D_glcm_JointEntropy | 0.998 |
| log-sigma-5-0-mm-3D_glcm_Idm | 0.997 |
| log-sigma-5-0-mm-3D_glcm_Idmn | 0.992 |
| log-sigma-5-0-mm-3D_glcm_Id | 0.996 |
| log-sigma-5-0-mm-3D_glcm_InverseVariance | 0.992 |
| log-sigma-5-0-mm-3D_glcm_MaximumProbability | 0.977 |
| log-sigma-5-0-mm-3D_glrlm_GrayLevelNonUniformityNormalized | 0.994 |
| log-sigma-5-0-mm-3D_glrlm_GrayLevelVariance | 0.999 |
| log-sigma-5-0-mm-3D_glrlm_HighGrayLevelRunEmphasis | 0.995 |
| log-sigma-5-0-mm-3D_glrlm_LongRunHighGrayLevelEmphasis | 0.912 |
| log-sigma-5-0-mm-3D_glrlm_LongRunLowGrayLevelEmphasis | 0.935 |
| log-sigma-5-0-mm-3D_glrlm_RunLengthNonUniformity | 0.873 |
| log-sigma-5-0-mm-3D_glrlm_RunLengthNonUniformityNormalized | 0.991 |
| log-sigma-5-0-mm-3D_glrlm_RunPercentage | 0.986 |
| log-sigma-5-0-mm-3D_glrlm_ShortRunEmphasis | 0.995 |
| log-sigma-5-0-mm-3D_glrlm_ShortRunHighGrayLevelEmphasis | 0.997 |
| log-sigma-5-0-mm-3D_glszm_GrayLevelNonUniformityNormalized | 0.991 |
| log-sigma-5-0-mm-3D_glszm_GrayLevelVariance | 0.978 |
| log-sigma-5-0-mm-3D_glszm_HighGrayLevelZoneEmphasis | 0.998 |
| log-sigma-5-0-mm-3D_glszm_SizeZoneNonUniformity | 0.958 |
| log-sigma-5-0-mm-3D_glszm_SizeZoneNonUniformityNormalized | 0.956 |
| log-sigma-5-0-mm-3D_glszm_SmallAreaLowGrayLevelEmphasis | 0.993 |
| log-sigma-5-0-mm-3D_glszm_ZoneEntropy | 0.998 |
| log-sigma-5-0-mm-3D_glszm_ZonePercentage | 0.937 |
| log-sigma-5-0-mm-3D_gldm_DependenceEntropy | 0.985 |
| log-sigma-5-0-mm-3D_gldm_DependenceNonUniformityNormalized | 0.986 |
| log-sigma-5-0-mm-3D_gldm_DependenceVariance | 0.974 |
| log-sigma-5-0-mm-3D_gldm_LargeDependenceEmphasis | 0.984 |
| log-sigma-5-0-mm-3D_gldm_LargeDependenceLowGrayLevelEmphasis | 0.930 |
| log-sigma-5-0-mm-3D_gldm_LowGrayLevelEmphasis | 0.959 |
| log-sigma-5-0-mm-3D_gldm_SmallDependenceHighGrayLevelEmphasis | 0.997 |
| log-sigma-5-0-mm-3D_gldm_SmallDependenceLowGrayLevelEmphasis | 0.983 |
| wavelet-LLH_firstorder_Entropy | 0.977 |
| wavelet-LLH_firstorder_InterquartileRange | 0.936 |
| wavelet-LLH_firstorder_Maximum | 0.837 |
| wavelet-LLH_firstorder_Mean | 0.823 |
| wavelet-LLH_firstorder_Median | 0.858 |
| wavelet-LLH_firstorder_RobustMeanAbsoluteDeviation | 0.945 |
| wavelet-LLH_firstorder_Skewness | 0.987 |
| wavelet-LLH_firstorder_Uniformity | 0.977 |
| wavelet-LLH_glcm_Autocorrelation | 0.824 |
| wavelet-LLH_glcm_JointAverage | 0.901 |
| wavelet-LLH_glcm_ClusterTendency | 0.955 |
| wavelet-LLH_glcm_Contrast | 0.906 |
| wavelet-LLH_glcm_DifferenceAverage | 0.955 |
| wavelet-LLH_glcm_DifferenceVariance | 0.835 |
| wavelet-LLH_glcm_JointEnergy | 0.989 |
| wavelet-LLH_glcm_Imc2 | 0.958 |
| wavelet-LLH_glcm_Idm | 0.965 |
| wavelet-LLH_glcm_Idmn | 0.915 |
| wavelet-LLH_glcm_Idn | 0.865 |
| wavelet-LLH_glcm_InverseVariance | 0.955 |
| wavelet-LLH_glcm_SumSquares | 0.947 |
| wavelet-LLH_glrlm_GrayLevelNonUniformityNormalized | 0.978 |
| wavelet-LLH_glrlm_GrayLevelVariance | 0.953 |
| wavelet-LLH_glrlm_LongRunEmphasis | 0.954 |
| wavelet-LLH_glrlm_LongRunHighGrayLevelEmphasis | 0.816 |
| wavelet-LLH_glrlm_RunEntropy | 0.984 |
| wavelet-LLH_glrlm_RunLengthNonUniformityNormalized | 0.941 |
| wavelet-LLH_glrlm_RunPercentage | 0.942 |
| wavelet-LLH_glrlm_ShortRunEmphasis | 0.951 |
| wavelet-LLH_glrlm_ShortRunHighGrayLevelEmphasis | 0.827 |
| wavelet-LLH_glszm_GrayLevelNonUniformityNormalized | 0.977 |
| wavelet-LLH_glszm_GrayLevelVariance | 0.938 |
| wavelet-LLH_glszm_HighGrayLevelZoneEmphasis | 0.822 |
| wavelet-LLH_glszm_LargeAreaHighGrayLevelEmphasis | 0.988 |
| wavelet-LLH_glszm_LargeAreaLowGrayLevelEmphasis | 0.996 |
| wavelet-LLH_glszm_SizeZoneNonUniformityNormalized | 0.902 |
| wavelet-LLH_glszm_SmallAreaEmphasis | 0.906 |
| wavelet-LLH_glszm_SmallAreaHighGrayLevelEmphasis | 0.839 |
| wavelet-LLH_glszm_ZoneEntropy | 0.974 |
| wavelet-LLH_glszm_ZonePercentage | 0.919 |
| wavelet-LLH_gldm_DependenceEntropy | 0.947 |
| wavelet-LLH_gldm_DependenceNonUniformityNormalized | 0.872 |
| wavelet-LLH_gldm_DependenceVariance | 0.955 |
| wavelet-LLH_gldm_GrayLevelVariance | 0.951 |
| wavelet-LLH_gldm_LargeDependenceEmphasis | 0.956 |
| wavelet-LLH_gldm_LargeDependenceHighGrayLevelEmphasis | 0.831 |
| wavelet-LLH_gldm_SmallDependenceEmphasis | 0.934 |
| wavelet-LLH_gldm_SmallDependenceHighGrayLevelEmphasis | 0.870 |
| wavelet-LLH_gldm_SmallDependenceLowGrayLevelEmphasis | 0.925 |
| wavelet-LHL_firstorder_90Percentile | 0.979 |
| wavelet-LHL_firstorder_Entropy | 0.975 |
| wavelet-LHL_firstorder_MeanAbsoluteDeviation | 0.957 |
| wavelet-LHL_firstorder_Mean | 0.823 |
| wavelet-LHL_firstorder_Median | 0.839 |
| wavelet-LHL_firstorder_RobustMeanAbsoluteDeviation | 0.938 |
| wavelet-LHL_firstorder_RootMeanSquared | 0.830 |
| wavelet-LHL_glcm_ClusterProminence | 0.962 |
| wavelet-LHL_glcm_ClusterShade | 0.935 |
| wavelet-LHL_glcm_ClusterTendency | 0.962 |
| wavelet-LHL_glcm_Correlation | 0.854 |
| wavelet-LHL_glcm_DifferenceAverage | 0.955 |
| wavelet-LHL_glcm_JointEnergy | 0.991 |
| wavelet-LHL_glcm_JointEntropy | 0.991 |
| wavelet-LHL_glcm_Imc1 | 0.814 |
| wavelet-LHL_glcm_Idm | 0.955 |
| wavelet-LHL_glcm_Id | 0.956 |
| wavelet-LHL_glcm_SumEntropy | 0.979 |
| wavelet-LHL_glcm_SumSquares | 0.961 |
| wavelet-LHL_glrlm_GrayLevelNonUniformityNormalized | 0.973 |
| wavelet-LHL_glrlm_LongRunEmphasis | 0.924 |
| wavelet-LHL_glrlm_LongRunLowGrayLevelEmphasis | 0.929 |
| wavelet-LHL_glrlm_RunLengthNonUniformityNormalized | 0.921 |
| wavelet-LHL_glrlm_RunPercentage | 0.919 |
| wavelet-LHL_glrlm_RunVariance | 0.915 |
| wavelet-LHL_glrlm_ShortRunLowGrayLevelEmphasis | 0.902 |
| wavelet-LHL_glszm_GrayLevelNonUniformityNormalized | 0.992 |
| wavelet-LHL_glszm_LargeAreaHighGrayLevelEmphasis | 0.963 |
| wavelet-LHL_glszm_LargeAreaLowGrayLevelEmphasis | 0.960 |
| wavelet-LHL_glszm_LowGrayLevelZoneEmphasis | 0.929 |
| wavelet-LHL_glszm_SizeZoneNonUniformity | 0.856 |
| wavelet-LHL_glszm_SizeZoneNonUniformityNormalized | 0.956 |
| wavelet-LHL_glszm_SmallAreaEmphasis | 0.961 |
| wavelet-LHL_glszm_ZoneEntropy | 0.984 |
| wavelet-LHL_glszm_ZonePercentage | 0.882 |
| wavelet-LHL_glszm_ZoneVariance | 0.972 |
| wavelet-LHL_gldm_DependenceNonUniformity | 0.803 |
| wavelet-LHL_gldm_DependenceNonUniformityNormalized | 0.867 |
| wavelet-LHL_gldm_LargeDependenceEmphasis | 0.934 |
| wavelet-LHL_gldm_LargeDependenceLowGrayLevelEmphasis | 0.978 |
| wavelet-LHL_gldm_LowGrayLevelEmphasis | 0.905 |
| wavelet-LHL_gldm_SmallDependenceHighGrayLevelEmphasis | 0.869 |
| wavelet-LHL_gldm_SmallDependenceLowGrayLevelEmphasis | 0.826 |
| wavelet-LHH_firstorder_Entropy | 0.982 |
| wavelet-LHH_firstorder_InterquartileRange | 0.944 |
| wavelet-LHH_firstorder_Kurtosis | 0.940 |
| wavelet-LHH_firstorder_MeanAbsoluteDeviation | 0.971 |
| wavelet-LHH_firstorder_Minimum | 0.972 |
| wavelet-LHH_firstorder_RootMeanSquared | 0.801 |
| wavelet-LHH_firstorder_Skewness | 0.894 |
| wavelet-LHH_firstorder_Uniformity | 0.973 |
| wavelet-LHH_glcm_Autocorrelation | 0.965 |
| wavelet-LHH_glcm_JointAverage | 0.964 |
| wavelet-LHH_glcm_ClusterTendency | 0.967 |
| wavelet-LHH_glcm_Contrast | 0.965 |
| wavelet-LHH_glcm_Correlation | 0.937 |
| wavelet-LHH_glcm_DifferenceEntropy | 0.978 |
| wavelet-LHH_glcm_DifferenceVariance | 0.972 |
| wavelet-LHH_glcm_Imc1 | 0.877 |
| wavelet-LHH_glcm_Imc2 | 0.875 |
| wavelet-LHH_glcm_Idm | 0.951 |
| wavelet-LHH_glcm_Id | 0.952 |
| wavelet-LHH_glcm_Idn | 0.870 |
| wavelet-LHH_glcm_SumEntropy | 0.981 |
| wavelet-LHH_glcm_SumSquares | 0.966 |
| wavelet-LHH_glrlm_GrayLevelNonUniformityNormalized | 0.981 |
| wavelet-LHH_glrlm_HighGrayLevelRunEmphasis | 0.967 |
| wavelet-LHH_glrlm_LongRunEmphasis | 0.895 |
| wavelet-LHH_glrlm_LowGrayLevelRunEmphasis | 0.942 |
| wavelet-LHH_glrlm_RunEntropy | 0.990 |
| wavelet-LHH_glrlm_RunLengthNonUniformityNormalized | 0.892 |
| wavelet-LHH_glrlm_RunVariance | 0.886 |
| wavelet-LHH_glrlm_ShortRunEmphasis | 0.900 |
| wavelet-LHH_glszm_GrayLevelNonUniformity | 0.816 |
| wavelet-LHH_glszm_GrayLevelNonUniformityNormalized | 0.955 |
| wavelet-LHH_glszm_GrayLevelVariance | 0.988 |
| wavelet-LHH_glszm_LowGrayLevelZoneEmphasis | 0.936 |
| wavelet-LHH_glszm_SizeZoneNonUniformity | 0.864 |
| wavelet-LHH_glszm_SmallAreaHighGrayLevelEmphasis | 0.965 |
| wavelet-LHH_glszm_SmallAreaLowGrayLevelEmphasis | 0.932 |
| wavelet-LHH_glszm_ZoneEntropy | 0.980 |
| wavelet-LHH_gldm_DependenceEntropy | 0.981 |
| wavelet-LHH_gldm_DependenceNonUniformityNormalized | 0.850 |
| wavelet-LHH_gldm_HighGrayLevelEmphasis | 0.967 |
| wavelet-LHH_gldm_LargeDependenceEmphasis | 0.896 |
| wavelet-LHH_gldm_LargeDependenceHighGrayLevelEmphasis | 0.882 |
| wavelet-LHH_gldm_LowGrayLevelEmphasis | 0.943 |
| wavelet-LHH_gldm_SmallDependenceEmphasis | 0.874 |
| wavelet-HLL_firstorder_10Percentile | 0.941 |
| wavelet-HLL_firstorder_90Percentile | 0.963 |
| wavelet-HLL_firstorder_Entropy | 0.956 |
| wavelet-HLL_firstorder_Kurtosis | 0.926 |
| wavelet-HLL_firstorder_Maximum | 0.967 |
| wavelet-HLL_firstorder_Median | 0.841 |
| wavelet-HLL_firstorder_Range | 0.875 |
| wavelet-HLL_firstorder_RobustMeanAbsoluteDeviation | 0.947 |
| wavelet-HLL_firstorder_Uniformity | 0.961 |
| wavelet-HLL_firstorder_Variance | 0.947 |
| wavelet-HLL_glcm_Contrast | 0.971 |
| wavelet-HLL_glcm_Correlation | 0.825 |
| wavelet-HLL_glcm_DifferenceAverage | 0.958 |
| wavelet-HLL_glcm_DifferenceVariance | 0.981 |
| wavelet-HLL_glcm_JointEnergy | 0.985 |
| wavelet-HLL_glcm_Imc2 | 0.934 |
| wavelet-HLL_glcm_Idm | 0.950 |
| wavelet-HLL_glcm_Idmn | 0.881 |
| wavelet-HLL_glcm_Idn | 0.874 |
| wavelet-HLL_glcm_InverseVariance | 0.955 |
| wavelet-HLL_glcm_SumSquares | 0.961 |
| wavelet-HLL_glrlm_GrayLevelNonUniformityNormalized | 0.963 |
| wavelet-HLL_glrlm_GrayLevelVariance | 0.950 |
| wavelet-HLL_glrlm_LongRunLowGrayLevelEmphasis | 0.908 |
| wavelet-HLL_glrlm_LowGrayLevelRunEmphasis | 0.899 |
| wavelet-HLL_glrlm_RunPercentage | 0.925 |
| wavelet-HLL_glrlm_RunVariance | 0.950 |
| wavelet-HLL_glrlm_ShortRunEmphasis | 0.918 |
| wavelet-HLL_glszm_GrayLevelNonUniformity | 0.807 |
| wavelet-HLL_glszm_GrayLevelNonUniformityNormalized | 0.960 |
| wavelet-HLL_glszm_LargeAreaLowGrayLevelEmphasis | 0.843 |
| wavelet-HLL_glszm_LowGrayLevelZoneEmphasis | 0.943 |
| wavelet-HLL_glszm_SizeZoneNonUniformity | 0.833 |
| wavelet-HLL_glszm_ZoneEntropy | 0.971 |
| wavelet-HLL_glszm_ZonePercentage | 0.886 |
| wavelet-HLL_gldm_DependenceNonUniformity | 0.814 |
| wavelet-HLL_gldm_DependenceNonUniformityNormalized | 0.910 |
| wavelet-HLL_gldm_DependenceVariance | 0.975 |
| wavelet-HLL_gldm_LargeDependenceEmphasis | 0.953 |
| wavelet-HLL_gldm_LargeDependenceLowGrayLevelEmphasis | 0.949 |
| wavelet-HLL_gldm_SmallDependenceLowGrayLevelEmphasis | 0.898 |
| wavelet-HLH_firstorder_10Percentile | 0.976 |
| wavelet-HLH_firstorder_90Percentile | 0.974 |
| wavelet-HLH_firstorder_InterquartileRange | 0.947 |
| wavelet-HLH_firstorder_Kurtosis | 0.907 |
| wavelet-HLH_firstorder_Mean | 0.846 |
| wavelet-HLH_firstorder_Median | 0.844 |
| wavelet-HLH_firstorder_Minimum | 0.980 |
| wavelet-HLH_firstorder_RobustMeanAbsoluteDeviation | 0.950 |
| wavelet-HLH_firstorder_RootMeanSquared | 0.877 |
| wavelet-HLH_firstorder_Variance | 0.988 |
| wavelet-HLH_glcm_Autocorrelation | 0.971 |
| wavelet-HLH_glcm_JointAverage | 0.969 |
| wavelet-HLH_glcm_ClusterShade | 0.913 |
| wavelet-HLH_glcm_ClusterTendency | 0.983 |
| wavelet-HLH_glcm_DifferenceAverage | 0.978 |
| wavelet-HLH_glcm_DifferenceEntropy | 0.994 |
| wavelet-HLH_glcm_DifferenceVariance | 0.995 |
| wavelet-HLH_glcm_JointEntropy | 0.991 |
| wavelet-HLH_glcm_Imc1 | 0.902 |
| wavelet-HLH_glcm_Idmn | 0.915 |
| wavelet-HLH_glcm_Id | 0.968 |
| wavelet-HLH_glcm_Idn | 0.886 |
| wavelet-HLH_glcm_MaximumProbability | 0.974 |
| wavelet-HLH_glcm_SumEntropy | 0.990 |
| wavelet-HLH_glrlm_GrayLevelVariance | 0.993 |
| wavelet-HLH_glrlm_HighGrayLevelRunEmphasis | 0.972 |
| wavelet-HLH_glrlm_LongRunEmphasis | 0.946 |
| wavelet-HLH_glrlm_LongRunLowGrayLevelEmphasis | 0.969 |
| wavelet-HLH_glrlm_LowGrayLevelRunEmphasis | 0.964 |
| wavelet-HLH_glrlm_RunPercentage | 0.925 |
| wavelet-HLH_glrlm_RunVariance | 0.939 |
| wavelet-HLH_glrlm_ShortRunEmphasis | 0.939 |
| wavelet-HLH_glrlm_ShortRunLowGrayLevelEmphasis | 0.963 |
| wavelet-HLH_glszm_GrayLevelNonUniformity | 0.839 |
| wavelet-HLH_glszm_HighGrayLevelZoneEmphasis | 0.980 |
| wavelet-HLH_glszm_LowGrayLevelZoneEmphasis | 0.965 |
| wavelet-HLH_glszm_SizeZoneNonUniformity | 0.920 |
| wavelet-HLH_glszm_SmallAreaHighGrayLevelEmphasis | 0.970 |
| wavelet-HLH_glszm_SmallAreaLowGrayLevelEmphasis | 0.985 |
| wavelet-HLH_gldm_DependenceEntropy | 0.979 |
| wavelet-HLH_gldm_DependenceNonUniformityNormalized | 0.846 |
| wavelet-HLH_gldm_DependenceVariance | 0.953 |
| wavelet-HLH_gldm_HighGrayLevelEmphasis | 0.972 |
| wavelet-HLH_gldm_LargeDependenceEmphasis | 0.948 |
| wavelet-HLH_gldm_LowGrayLevelEmphasis | 0.964 |
| wavelet-HLH_gldm_SmallDependenceEmphasis | 0.871 |
| wavelet-HLH_gldm_SmallDependenceHighGrayLevelEmphasis | 0.959 |
| wavelet-HHL_firstorder_10Percentile | 0.944 |
| wavelet-HHL_firstorder_90Percentile | 0.969 |
| wavelet-HHL_firstorder_Maximum | 0.943 |
| wavelet-HHL_firstorder_MeanAbsoluteDeviation | 0.965 |
| wavelet-HHL_firstorder_Minimum | 0.879 |
| wavelet-HHL_firstorder_RobustMeanAbsoluteDeviation | 0.955 |
| wavelet-HHL_firstorder_Skewness | 0.972 |
| wavelet-HHL_glcm_Autocorrelation | 0.861 |
| wavelet-HHL_glcm_JointAverage | 0.887 |
| wavelet-HHL_glcm_ClusterProminence | 0.893 |
| wavelet-HHL_glcm_Contrast | 0.972 |
| wavelet-HHL_glcm_Correlation | 0.893 |
| wavelet-HHL_glcm_DifferenceVariance | 0.974 |
| wavelet-HHL_glcm_JointEnergy | 0.955 |
| wavelet-HHL_glcm_JointEntropy | 0.985 |
| wavelet-HHL_glcm_Imc2 | 0.953 |
| wavelet-HHL_glcm_Idm | 0.951 |
| wavelet-HHL_glcm_Idn | 0.822 |
| wavelet-HHL_glcm_InverseVariance | 0.968 |
| wavelet-HHL_glcm_MaximumProbability | 0.944 |
| wavelet-HHL_glcm_SumEntropy | 0.983 |
| wavelet-HHL_glcm_SumSquares | 0.971 |
| wavelet-HHL_glrlm_GrayLevelNonUniformityNormalized | 0.965 |
| wavelet-HHL_glrlm_HighGrayLevelRunEmphasis | 0.867 |
| wavelet-HHL_glrlm_LongRunEmphasis | 0.852 |
| wavelet-HHL_glrlm_LowGrayLevelRunEmphasis | 0.917 |
| wavelet-HHL_glrlm_RunEntropy | 0.990 |
| wavelet-HHL_glrlm_RunLengthNonUniformityNormalized | 0.873 |
| wavelet-HHL_glrlm_RunVariance | 0.847 |
| wavelet-HHL_glrlm_ShortRunEmphasis | 0.873 |
| wavelet-HHL_glszm_GrayLevelNonUniformity | 0.808 |
| wavelet-HHL_glszm_GrayLevelNonUniformityNormalized | 0.944 |
| wavelet-HHL_glszm_GrayLevelVariance | 0.917 |
